# Effect of soft tissue sample preparation techniques for scanning small-angle X-ray scattering experiments

**DOI:** 10.1107/S1600577526001530

**Published:** 2026-04-14

**Authors:** Atreyee Acharya, Arthur Baroni, Irene Rodriguez-Fernandez, Mads Carlsen, Marianne Liebi

**Affiliations:** a Center for Photon Science Paul Scherrer Institut Villigen Switzerland; b Institute of Materials École Polytechnique Fédérale de Lausanne Lausanne Switzerland; c Institute for Biomedical Engineering ETH Zürich Zürich Switzerland; Forschungszentrum Jülich, Germany

**Keywords:** small-angle X-ray scattering, fixation, embedding, collagen structure, muscle structure

## Abstract

Scanning small-angle X-ray scattering (sSAXS) has found multiple applications as a technique to probe the nanostructure in soft tissues and pathologies thereof. However, fresh tissue is fragile and prone to the quick onset of decomposition and autolysis. It lacks the firmness required for uniform and thin sectioning, resulting in the loss of 2D resolution offered by focused X-ray beams, because the signal would be integrated through thick and/or irregular sections. Tissue processing, that includes fixation and embedding, is used to mitigate these issues but can by itself introduce structural changes in the tissues and impede the correct interpretation of sSAXS data. Here the extent of these structural changes in the SAXS signal caused by common tissue preservation methods on the example of skeletal muscle tissue, consisting of both muscle and surrounding connective tissue, was studied. This can guide an informed choice of preservation method tailored for specific experimental requirements. While some techniques per­formed better than others, all tissue-processing methods induced structural changes to a certain degree. The choice of preservation method is therefore a balance between sectioning requirements and type of tissue used, as well as targeted structural information.

## Abstract 2

The effect of tissue-processing techniques, including fixation and embedding, on the structural information obtained from scanning small-angle X-ray scattering of muscle and connective tissue has been studied. This provides a framework for sSAXS users working with soft tissues to make an informed choice on the sample preservation method, tailored to their own requirements.

## 1. Introduction

Small-angle X-ray scattering (SAXS) is an effective technique to characterize nanostructure, statistically averaged over the X-ray illuminated sample volume. SAXS is used for a wide range of materials and can be used to retrieve the morphology of nanostructured materials, such as size and shape, or, in ordered systems, structural information on nanoscale lattice periodicities (Glatter & Kratky, 1982 ▸; Guinier *et al.*, 1955 ▸; Feigin & Svergun, 1987 ▸). In soft tissues, SAXS is a promising tool to study supramolecular structure and detect structural changes in the tissue and how it relates to the pathology of various diseases (Lewis *et al.*, 2000 ▸; Malinchik *et al.*, 1998 ▸). The periodic nature of well-ordered supramolecular structures results in distinct diffraction peaks, such as from the regular *D*-period along the collagen fibril (Bear, 1942 ▸), the myo­fibrillar lattice structure in different muscle tissues (Iwamoto *et al.*, 2002 ▸) and repeated myelination around neurons (Georgiadis *et al.*, 2021 ▸). By scanning the sample area with a focusedX-ray beam, *i.e.* scanning SAXS (sSAXS), it is possible to obtain the variation of these nanoscale structural and their orientational parameters over a macroscopic scale. With advancements in large-scale synchrotron facilities – offering a large photon flux, smaller beam sizes and high frame rate X-ray detectors – high-resolution measurements with short acquisition times have become feasible (Westneat *et al.*, 2008 ▸; Müller *et al.*, 2010 ▸). This makes it possible to bridge the gap between the macroscopic tissue dimensions and variability, and the changing supramolecular structures (Bunk *et al.*, 2009 ▸). As examples of soft tissue applications, synchrotron scanning imaging techniques using scattering or X-ray fluorescence contrast have been used to examine collagen structural changes in breast cancer (Fernández *et al.*, 2005 ▸; Conceição *et al.*, 2009 ▸), to probe microcalcifications in tumours and aortic diseases (Arboleda *et al.*, 2019 ▸; Giannini *et al.*, 2019 ▸), to analyze structural differences in neurodegenerative diseases (Müller *et al.*, 2010 ▸; Solvas *et al.*, 2019 ▸; Choi *et al.*, 2020 ▸; Carboni *et al.*, 2017 ▸), and many other soft tissue dis­orders (Castro *et al.*, 2005 ▸).

One hurdle to accurate data interpretation is structural changes induced by tissue-processing methods. Processing and fixation of tissue are essential to preserve tissue in a near-native state by preventing autolysis and putrefaction. For many synchrotron sources, it is also necessary to inactivate biological tissues, particularly those derived from human pathology, to comply with the required biosafety standards of many beamlines. Additionally, tissues can be infiltrated by an embedding medium to facilitate thin sectioning of tissues and long-term preservation. However, these processing techniques, including physical, chemical and thermal processes, can alter the structure of tissue components and, in the presence of chemical and embedding media, can interfere with the X-ray scattering signals through overlapping background scattering.

The aim of our study was to assess the effects of a comprehensive list of preservatives and embedding media on the native structure of typical soft tissues using sSAXS. This would guide the selection of preservation technique for future soft tissue experiments, and raise awareness on how scattering features are changing, towards cautious interpretation of the results. Bovine skeletal muscle, including connective tissue, was chosen as the test sample for this experiment, as it allows simultaneous observation of the changes to the characteristic SAXS signal of muscle and collagen tissue components without posing safety issues for measuring non-fixed reference samples at the synchrotron beamline.

### 1.1. Structure of skeletal muscle

**Figure 1:**
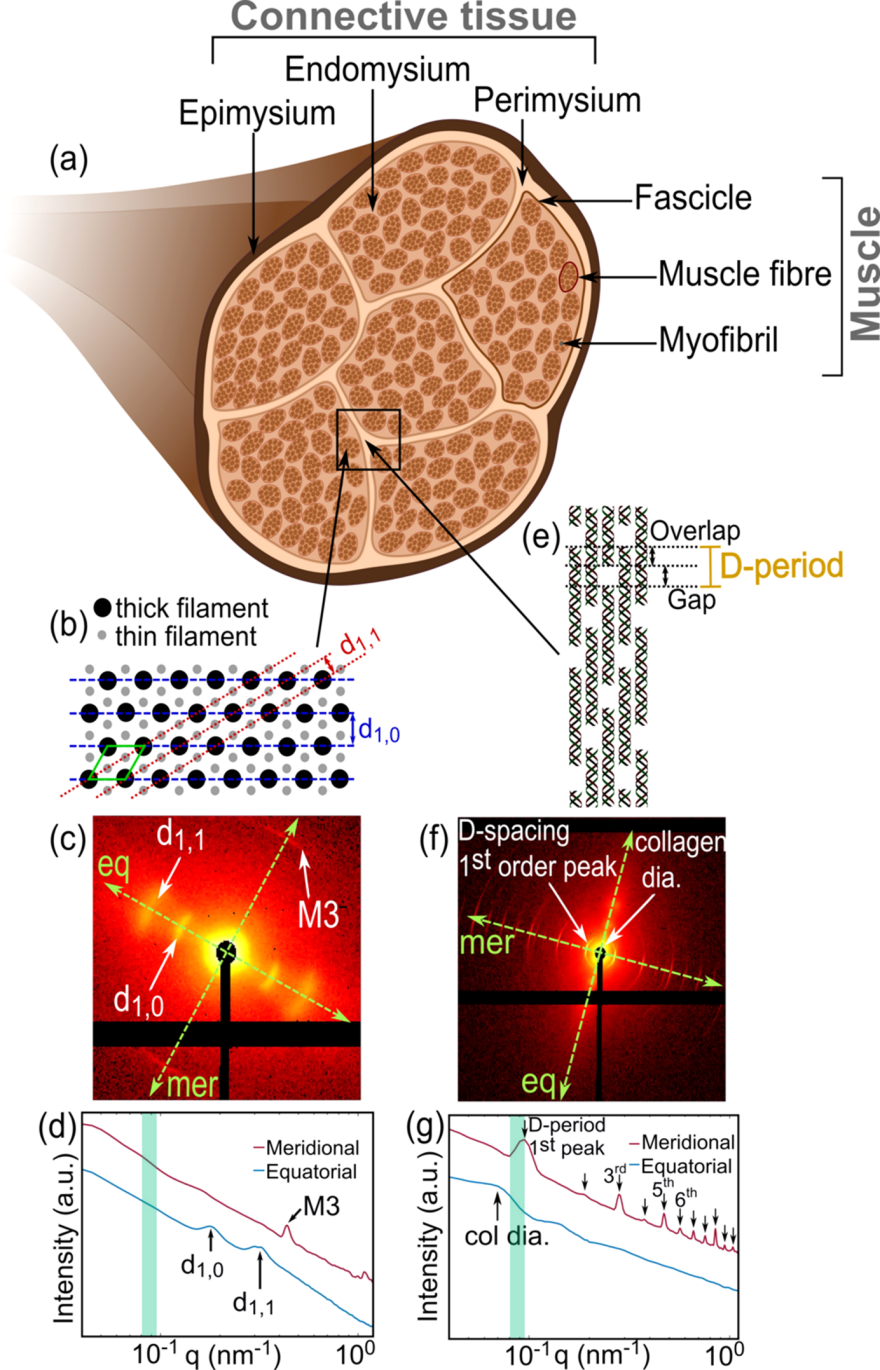
(*a*) Anatomy of skeletal muscle. (*b*) The cross-sectional packing of skeletal myofilaments exhibiting the hexagonal lattice structure with lattice planes 1,0 (blue dashed lines) and 1,1 (red dotted lines). The unit cell is shown in green. (*c*) Typical SAXS pattern of muscle with visible equatorial features corresponding to the interplanar distances in part (*b*). M3 is the third meridional reflection arising from the quasi-helical arrangement of myosin heads protruding from the cylindrical thick filament structure (Ma & Irving, 2022 ▸). (*d*) Scattering intensity distribution of myo­fibril arrangement in the meridional (mer) and equatorial (eq) directions, obtained by integrating intensities in part (*c*), as a function of scattering vector, *q*. (*e*) The lateral structural organization of a collagen fibril in perimysium, made of individual collagen molecules. (*f*) A typical collagen SAXS pattern showing several orders of meridional peaks corresponding to the *D*-period noted in part (*e*). The broad equatorial scattering, perpendicular to the meridional direction, containing information about the transverse direction, including fiber diameter. (*g*) Scattering intensity distribution of collagen fiber arrangement in the meridional (mer) and equatorial (eq) directions, obtained by integrating intensities in part (*f*) as a function of scattering vector, *q*. The green highlighted regions of the plots in parts (*d*) and (*g*) have been utilized for segmentation of regions, which is explained further in Section 2.3.1.

Skeletal muscle is composed of muscle fibers surrounded by intramuscular connective tissue, arranged hierarchically [Fig. 1 ▸(*a*)]. The smallest functional unit of a muscle, called a sarcomere, includes a thick filament made of myosin and a thin filament composed of actin, whose relative sliding movement enables the contraction of muscles. Multiple sarcomeres form a myo­fibril in an end-to-end arrangement, and several myo­fibrils are arranged in parallel along the long axis to form a muscle fiber. Many of these fibers bundle together to form a single unit of fascicle and several fascicles are arranged cylindrically to constitute muscle tissue. Each unit of muscle fiber and fascicle, as well as each individual muscle tissue, is surrounded by connective tissue structures called endomysium, perimysium and epimysium, respectively, together making up the intramuscular connective tissue (Mukund & Subramaniam, 2020 ▸).

The cross-section of a muscle fiber resembles a hexagonal lattice with a single unit cell comprising of one thick and two thin filaments [Fig. 1 ▸(*b*)]. This arrangement produces a prom­i­nent equatorial SAXS pattern [Fig. 1 ▸(*c*)], with two broad peaks [Fig. 1 ▸(*d*)] pertaining to the lattice interplanar spacings of the 1,0 and 1,1 crystallographic planes. In simplified terms, both thick and thin filaments contribute to the *I*_1,1_ scattered intensity, while only thick filaments are responsible for the *I*_1,0_ intensity. Changes in the equatorial ratio *I*_1,1_/*I*_1,0_ and the width of the scattering pattern have been associated with multiple muscle disorders (Ma & Irving, 2022 ▸). The meridional scattering pattern is due to the periodic structures along the length of the myosin, actin and other accessory proteins.

The main component of intramuscular connective tissue is collagen. It is the primary structural protein in the body categorized into 28 different types (Ricard-Blum, 2010 ▸), out of which collagen I is the most abundant. A collagen molecule is composed of three polypeptide chains, or α-chains, forming a triple helical structure. Fibrillar collagen (type I, II, III, V and XI) molecules assemble into fibrils, a process driven by telopeptides forming intermolecular cross-links (Shayegan *et al.*, 2016 ▸). These fibrils have a periodic axial banded structure, called the *D*-period, of the order ∼67 nm (Petruska & Hodge, 1964 ▸). The *D*-period is formed by the regular intervals of gap and overlap in the arrangement of collagen molecules [Fig. 1 ▸(*e*)], which transpire into diffraction peaks of several orders in the meridional direction [Figs. 1(*f*) ▸ and 1(*g*) ▸]. The equatorial scattering is caused by the packing of the collagen molecules into fibrils 50–200 nm in diameter (Fratzl, 2008 ▸).

### 1.2. Interaction of soft tissues with preservatives

To understand the differences between the structural mod­i­fi­ca­tions caused by the different preservatives, it is vital to consider the type of interactions that may occur between them and the soft tissues. This section focuses on methods utilized in this experiment and is therefore not an exhaustive list.

Physical fixation techniques, such as drying with silica gel, use non-chemical means of water removal, which stabilizes the tissue. Silica desiccant is mainly used as a preservative in plant tissues or, in certain cases, hard animal tissues. While it can absorb moisture inhibiting enzymatic activity to some extent, it can cause significant structural distortion in soft tissues due to dehydration-induced shrinkage (Nicoletti *et al.*, 2021 ▸). We would expect that a similar loss of structural rigidity is caused by another drying technique called SpeedVac. Here a centrifugal vacuum concentrator is used for rapidly dehydrating a small volume of samples using a combination of lowered pressure, centrifugal force and high temperature. Given the significantly larger size of tissue samples, causing the need for prolonged speed-vacuuming, as well as a higher temperature, at least a partial denaturation and loss of structure is to be expected.

Chemical fixation techniques include perfusing soft tissues with a chemical fixative solution to prevent autolysis. A 10% formalin solution is often used, containing ∼4% formaldehyde, which binds to amino acids in proteins and forms cross-links between them to stabilize the structure and prevent autolysis (Thavarajah *et al.*, 2012 ▸). Phosphate-buffered saline (PBS) solution is usually not used as a standalone long-term storage solution for tissues but as a short-term storage medium after previous chemical fixation. While it works to keep the tissues hydrated to maintain structural integrity, it does not prevent enzymatic degradation or microbial growth (Sengupta *et al.*, 2013 ▸; Wright *et al.*, 2014 ▸). Alcohols, like methanol and ethanol, act as dehydrating and precipitating fixation agents that disrupt the hydro­philic and hydro­phobic bonds in proteins and changes the tertiary structure, while keeping the triple helical structure of collagen intact (Turunen *et al.*, 2017 ▸; Haverkamp *et al.*, 2022 ▸; Xu *et al.*, 2021 ▸). A 10% formalin fixation followed by dehydration in ethanol are most often the initial steps in embedding procedures. RNA*later*, a solution containing ammonium sulfate buffered with sodium citrate and EDTA (ethyl­enedi­amine­tetra­acetic acid) that acts as a chelating agent, was developed to stabilize nucleic acids but has lately been investigated as a tissue preservative for histological analysis as well. The interaction mechanism between RNA*later* and tissues remains unclear and there are conflicting results about its effectiveness as a preservative for soft tissues (Nicoletti *et al.*, 2021 ▸; Wang *et al.*, 2018 ▸).

In cryopreservation techniques, enzymatic processes slow down due to ultra-low temperatures and the growth of microbes is prevented. However, even snap-freezing, or the rapid cooling of a sample to minimize ice damage, has been shown to cause mechanical damage and not preserve all morphological features well in certain cases (Kennedy *et al.*, 2007 ▸). Additionally, samples undergoing multiple freeze–thaw cycles would also change their structure (Ozcelikkale *et al.*, 2013 ▸). OCT (Optimum Cutting Temperature compound, Tissue-Tek) is a common embedding medium for cryopreservation that helps to stabilize the tissue and facilitate cryostat sectioning. OCT is made of heavy molecules like poly­vinyl alcohol (PVA) and poly­ethyl­ene glycol (PEG).

Embedding procedures are often coupled with chemical fixation to preserve tissue morphology and enable uniform sectioning. Paraffin embedding, which is preceded by formalin and ethanol fixation, is the gold standard for the histology of soft tissues. In addition to the dehydration and fixation steps, xylene, which is used as a solvent to replace the ethanol, and paraffin infiltration at high temperature (around 60°C) can have negative effects on the nanostructure, such as differential volumetric shrinkage, as well as a change in optical properties, which are not necessarily evident in regular histological analysis (Rodgers *et al.*, 2021 ▸; Azril *et al.*, 2023 ▸). Osmium-stained tough-resin-embedded tissues are used to enable correlative electron microscopy studies. While the main purpose of this staining is to enhance contrast by binding to lipids in tissues, it also stains collagen, altering the electron-density distribution that changes the relative intensities visualized during SAXS experiments (Lam *et al.*, 1978 ▸). The fixation, dehydration and staining steps coupled with shrinkage due to ep­oxy embedding has been shown to affect the tissue architecture (Brilakis *et al.*, 2001 ▸). The type of ep­oxy used is expected to change the results. With polymethyl­methacrylate (PMMA)-based embedding processes like Techno­vit 9100, there is also a risk of polymerization shrinkage (Orr & Dunne, 2004 ▸). However, the low-temperature polymerization process might help to mitigate that (Yang *et al.*, 2003 ▸). There are also several other components, like hardeners and regulators, involved in the embedding process that may have an effect on the structure, along with the pre-embedding steps involved.

Collagen structure has been shown to be especially sensitive to the dehydration process (Bigi *et al.*, 1987 ▸; Wess & Orgel, 2000 ▸). A study using SAXS reported that ethanol fixation severely affected the collagen ultrastructure, while formalin fixation did not cause any significant change (Turunen *et al.*, 2017 ▸). Alternatively, another study found that preserving corneal collagen in 10% formalin and other preservative solutions led to changes in the *D*-period of collagen, as well as the fibril diameter distribution (Kelly *et al.*, 2021 ▸). The interaction of collagen with an increasing concentration of propanol displayed that the dehydration of collagen occurs as a two-step process: an increase in the gap-to-overlap ratio, indicating a restructuring within the fibrils, while the *D*-period remains constant in the first step, and a decrease in the *D*-period itself in the second step (Haverkamp *et al.*, 2022 ▸).

Table 1 ▸ includes a non-exhaustive list of preservation techniques used in X-ray-based soft tissue studies in the literature. However, it is still unclear for most preservation techniques what effect they have on the observed SAXS signal.

## 2. Materials and methods

### 2.1. Sample and tissue-processing methods

**Figure 2:**
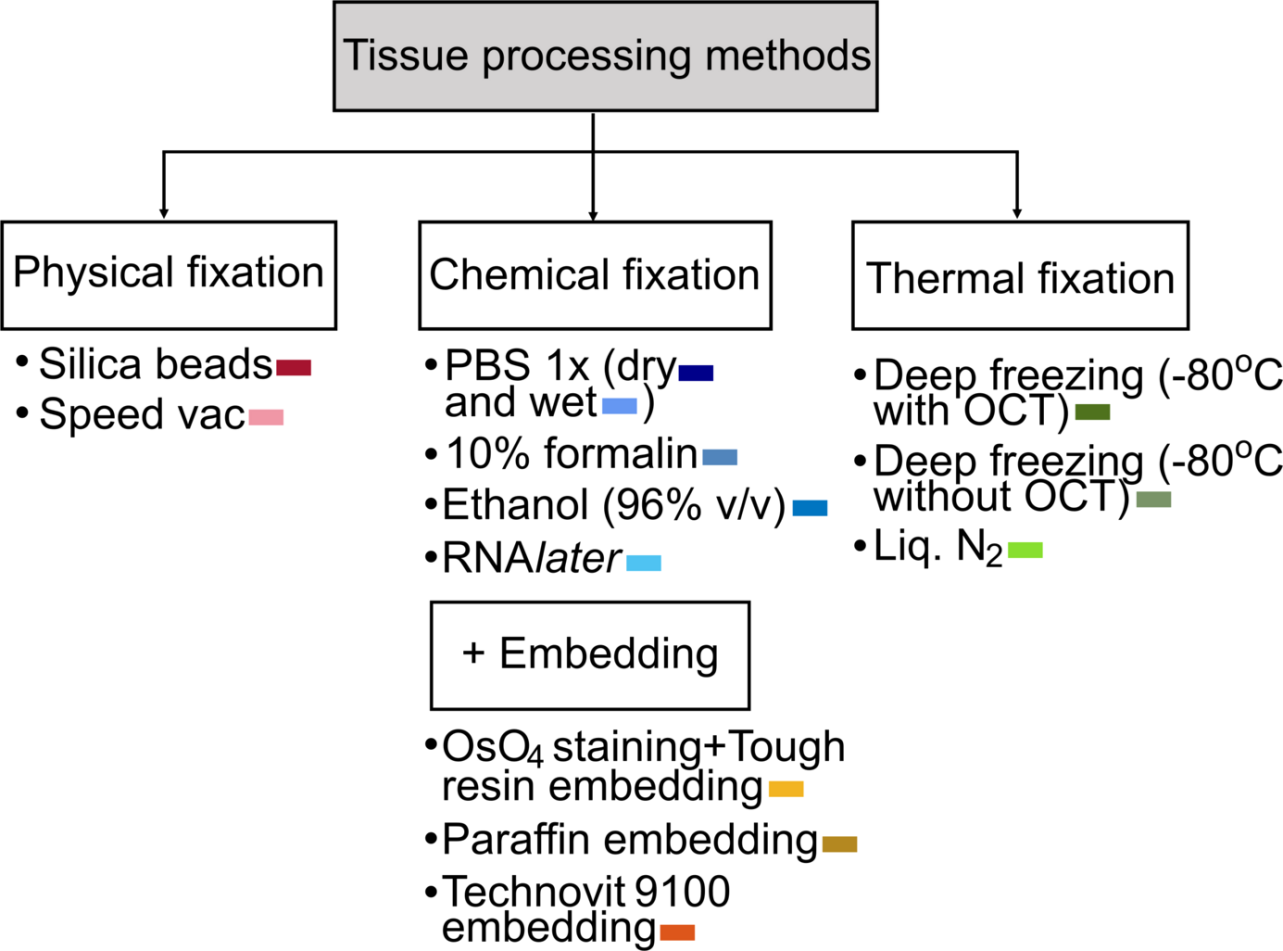
The different tissue-processing methods used in this study. The colour code indicated here has been followed for the respective samples throughout the article.

Packaged bovine meat was obtained from the refrigerated (4°C) section of the supermarket, sourced from the shank region of veal, which was chosen for its high connective tissue composition and skeletal muscle organization. The choice of sample was made based on the ease of availability and the lack of ethical and safety constraints for the experiment, while providing distinct scattering features from both muscle tissue and collagen. A wide variety of tissue-processing methods were probed (Fig. 2 ▸) ranging from elementary field-preservation methods, such as drying in silica-gel beads, short-term storage solutions, such as PBS, to more widespread techniques, such as 10% formalin fixation, paraffin embedding and cryopreservation techniques.

The meat was cut such that each piece for the different processing methods had the two visually distinct regions, a red region corresponding to skeletal muscle tissue and a white connective tissue region. All samples, except the embedded ones, were cut before processing using a sterile surgical grade scalpel into a 1 cm × 1 cm cross-sectional area of 1 mm-thickness. The embedded samples were sectioned differently, as facilitated by their embedding protocols. An unprocessed sample with no fixation, to be used as control, was refrigerated at 4°C for 72 h before measurement. The paraffin embedding was repeated on another sample derived from a different bovine meat block, since the logistics of the first experiment [denoted paraffin(I)] led to a prolonged storage in formalin and a longer transport time which affected sample morph­ol­ogy rather than the embedding process itself. The Technovit sample was sourced from yet another piece of bovine meat and an additional unprocessed sample as control from the new meat sample was refrigerated at 4°C overnight before meas­ure­ment. From each different bovine meat block a separate unprocessed sample was measured as reference. Besides 1 mm-thick embedded samples, 30 µm-thick tough-resin- and paraffin-embedded samples were prepared to understand the effect of tissue thickness on sSAXS results.

#### 2.1.1. Physical methods

(i) **Drying with silica-gel beads**. A 1 mm-thick sample was immersed in silica-gel beads in an enclosed tube and refrigerated at 4°C for 72 h. The beads were inspected regularly for colour change and replaced when no longer dry.

(ii) **Drying in a SpeedVac**. A 1 mm-thick sample was placed in an Eppendorf tube to be speed-vacuum dried, a centrifugal vacuum concentrator, at 40°C in vacuum conditions for 8 h. The retrieved sample was then placed in a separate enclosed tube and refrigerated at 4°C before measurement.

#### 2.1.2. Chemical methods

(i) **PBS**. Two 1 mm-thick samples were immersed in PBS 1x (ROTI Cell) solution in an enclosed tube and refrigerated at 4°C for 72 h. One was taken out of the PBS and measured in a dry environment and the second was immersed in PBS during measurement.

(ii) **Formalin**. A 1 mm-thick sample was immersed in 10% neutral-buffered formalin and placed in an enclosed tube at 4°C for 72 h.

(iii) **Ethanol**. A 1 mm-thick sample was immersed in 96% *v*/*v* ethanol solution in an enclosed tube and refrigerated at 4°C for 72 h.

(iv) **RNA*later***. A 1 mm-thick sample was immersed in RNA*later* stabilization solution (Ambion, Inc.) in an enclosed tube at 4°C for 72 h.

#### 2.1.3. Embedding methods

(i) **Osmium staining and tough-resin embedding**. Smaller separate sections of tissue samples, approximately 2 mm × 1 mm in cross-section and 1 mm-thick, containing either solely muscle or connective tissue region, were fixed using 10% formalin and stained with 2% osmium tetroxide (OsO_4_). They were then embedded in triglycidyl para-amino­phenol (TGPAP) resin, a trifunctional ep­oxy resin known for its thermal stability and radiation resistance and hardened using 4,4′-di­amino­diphenyl­methane (DDM) according to a protocol defined by Bosch *et al.* (2023 ▸), which included OsO_4_ staining. The method of ep­oxy embedding is commonly used in transmission electron microscopy (TEM) studies and utilizes smaller sections to ensure adequate fixation and infiltration. Additionally, two such samples, one each of muscle and connective tissue, were polished down to a thickness of 300 µm using abrasive papers.

(ii) **Paraffin embedding**. This embedding process was carried out by the Histology Core Facility at Swiss Federal Institute of Technology Lausanne (EPFL), Switzerland. The process involved a block of tissue (1 cm × 1 cm × 1 cm), 10% formalin fixed and dehydrated using 70% *v*/*v* ethanol, being embedded in paraffin and later scalpel-sectioned into a slice of around 1 mm thickness, in order to compare it directly with the non-embedded samples. Another section of 30 µm was cut from the block using a microtome. The two cuts were used to assess the effect of sample thickness in the experiment. Since logistical delays caused a longer storage time in formalin and ethanol, the process was repeated on a second set of samples (different source), both 1 mm and 30 µm thickness, in a more time-controlled manner. Henceforth, the first round of embedded samples have been abbreviated to paraffin(I) and the second to paraffin(II), with a separate control sample being denoted unprocessed(II).

(iii) **Technovit 9100 embedding**. A 1 mm-thick sample was fixed in 10% formalin and dehydrated progressively in 70, 96 and 100% *v*/*v* ethanol, followed by infiltration in xylene, that acts as an intermedium. The basis solution was used in stabilized form and the multi-step embedding process was carried out according to the Technovit 9100 (Kulzer GmbH, Germany) instruction manual. The reason for choosing this embedding is that the polymerization could occur below freezing temperatures to minimize the effect of heat of polymerization influencing the samples. Since this embedding was per­formed on a different meat source, the reference sample here is unprocessed(III), while another such sample was pretreated in the same manner in formalin, ethanol and xylene mentioned above, without the embedding, to solely understand the effect of embedding itself.

#### 2.1.4. Thermal (cryo) methods

(i) **Deep freezing (−80°C, no embedding medium)**. A 1 mm-thick tissue section was placed in a cryomold, snap frozen on dry ice and then stored in an ultra-cold freezer at −80°C for 72 h.

(ii) **Deep freezing with OCT embedding**. A 1 mm-thick sample was placed in a cryomold embedded in OCT medium (Tissue-Tek Optimum Cutting Temperature compound, Sakura Finetek, Inc.), followed by snap-freezing using dry ice and storage in an ultra-low-temperature freezer (−80°C) for 72 h.

(iii) **Liquid nitro­gen**. A 1 mm-thick tissue section was placed in a cryovial and stored in a liquid nitro­gen cryogenic storage dewar for 72 h.

Each sample, excluding the embedded ones, was placed in a pocket made of Kapton foil (25 µm thick on each side) and glued shut to prevent dehydration in air. Since the embedded samples were not at risk of dehydration, they were mounted between Kapton tape (13 µm thick) on both sides to hold them in place. All samples were fixed on a motorized sample plate before measurement. The cryopreserved samples could not be measured in a frozen state due to the unavailability of a cryostream at the beamline.

### 2.2. Scanning small-angle X-ray scattering (sSAXS)

**Figure 3:**
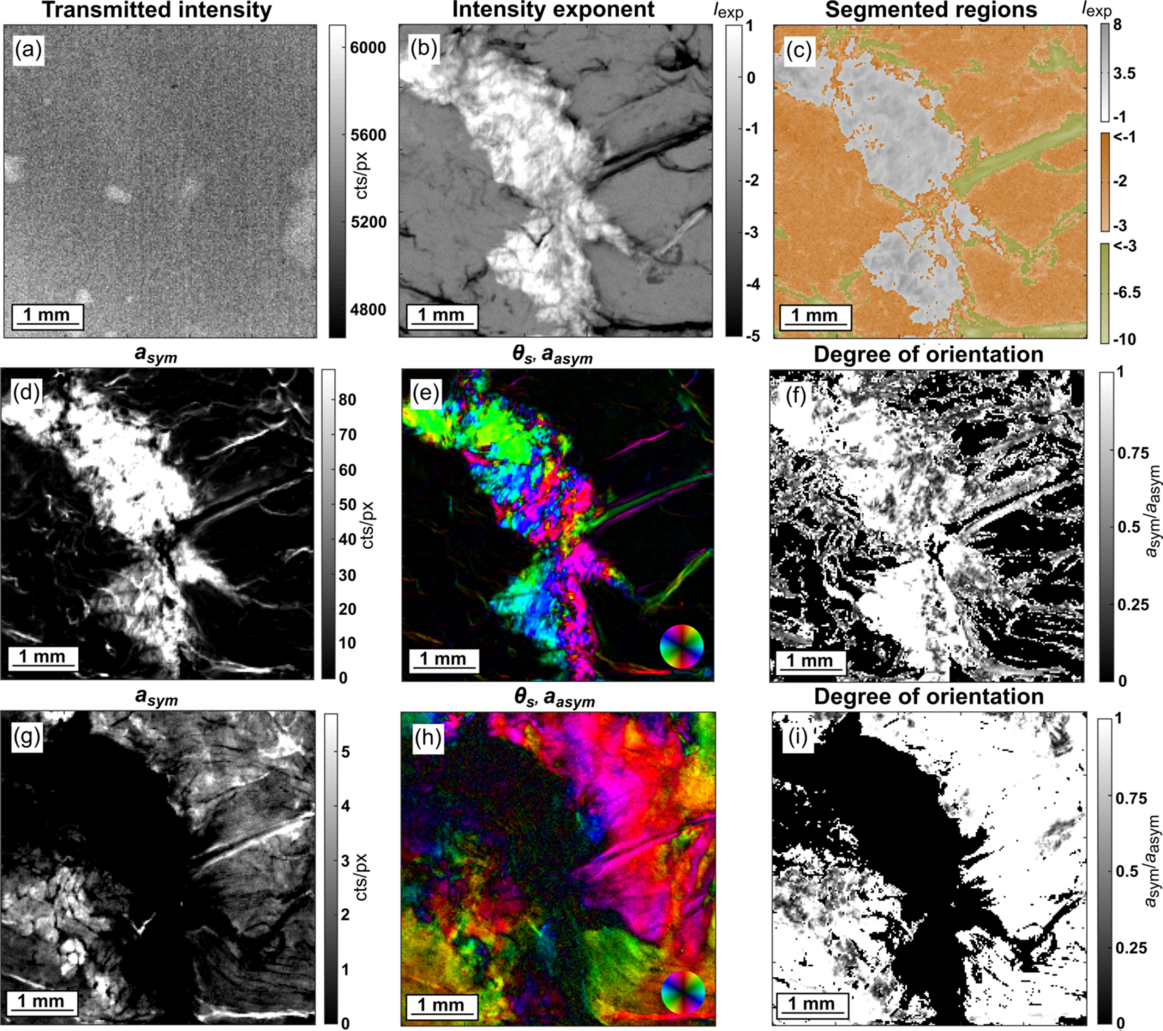
Scanning SAXS images of unprocessed (control) meat sample. Image plots showing (*a*) transmission, (*b*) intensity exponent in the range *q* = 0.085–0.095 nm^−1^, (*c*) colour-coded segmentation of regions based on intensity exponent variation in part (*b*), (*d*) symmetric intensity, (*e*) hue-value representation (HSV plot with saturation = 1) of orientation of meridional scattering (θ_s_, colourwheel) and asymmetric intensity (value) of scattering (*a*_asym_), and (*f*) degree of orientation, over the first-order collagen (meridional) peak with *q* = 0.085–0.11 nm^−1^. Parts (*g*)–(*i*) represent the same figures in the order of parts (*d*)–(*f*) over the *d*_1,1_ myo­fibril (equatorial) peak with *q* = 0.231–0.367 nm^−1^.

Scanning SAXS experiments were carried out at the cSAXS beamline at the Swiss Light Source at Paul Scherrer Institut, Villigen, Switzerland, during four beam times. The measured samples and the experimental parameters are detailed in Table S1 of the supporting information. Samples were raster scanned using an X-ray beam in a continuous line scanning mode, with the scanning step size being 25 µm in either direction. The scanning plate was positioned perpendicular to the direction of the X-ray beam with an evacuated 2 m-long flight tube in between to minimize air scattering, and an X-ray detector was positioned at the other end of the tube. An exposure time of 100 ms per scanning point was used to minimize radiation damage. The transmission was measured by the scattering from a 1.5 mm steel beamstop placed inside the flight tube [Fig. 3 ▸(*a*)]. This was later utilized for data normalization.

### 2.3. Data analysis

Data reduction and analysis was per­formed using the cSAXS *Matlab* package available for scanning SAXS (Bunk *et al.*, 2009 ▸). Data in each scan point were reduced by azimuthal integration into 16 angular segments and radial integration across the available *q*-range into ∼1200 data points.

#### 2.3.1. Segmentation of different regions and statistics

The different regions in the samples were segmented using the integrated intensity exponent (power-law exponent) in the *q*-range 0.085–0.095 nm^−1^ [highlighted in Figs. 1 ▸(*d*) and 1 ▸(*g*)], *i.e.* the region corresponding to the first half of the first-order collagen peak, as the slope of this region provides good contrast between the collagenous and non-collagenous regions. This contrast in the unprocessed sample [Fig. 3 ▸(*b*)] demonstrates the collagen region in white due to the initial positive slope of the first-order peak and two distinct regions with a negative intensity exponent, in gray and black, due to an absent collagen peak. The samples with an absent first-order collagen peak due to the processing method had an enhanced sixth-order meridional reflection, which was used to implement the same classification. Based on this analysis, the samples were segmented into three distinct regions [Fig. 3 ▸(*c*)], thresholded using the intensity exponent. Region 1 (gray) consists of collagen, while in region 2 the SAXS pattern was typical of myo­fibrils from the muscle (orange). We also identified a distinct third region (green) with an even lower intensity exponent, showing three broad SAXS peaks, located at approximately *q* = 0.075, 0.16 and 0.245 nm^−1^. The very low intensity exponent of this region is thus explained by the *q*-range being in the decaying part of the first broad peak. We surmise that this region corresponds to other supramolecular structures present in the connective tissue of skeletal muscle and we did not proceed with a detailed analysis of this region. The typical 1D plots of scattering intensity, *I*(*q*), against the scattering vector, *q*, for the three regions is shown in Fig. S2 in the supporting information. The regions classified as muscle and collagen, each comprising thousands of data points, were then used to identify and compare trends in the different regions within samples and evaluate the statistical differences between similar regions across samples.

These results are plotted in Section 3 to compare parameters like *D*-period and peak width for both collagen and myo­fibrils. The Wilcoxon signed-rank test was applied to assess the statistical differences in these parameters between samples. For instance, the *D*-period distribution of collagen in processed samples was compared against the same in a reference sample. The null hypothesis is that the distributions are the same and a *p*-value > 0.05 indicated a statistical difference between them.

#### 2.3.2. Orientation analysis

The variation of intensity across the azimuthal segments within a defined *q*-range of each scattering pattern was fitted using a cosine function to extract the following parameters (Bunk *et al.*, 2009 ▸): symmetric intensity of scattering (*a*_*sym*_) corresponding to the average scattering intensity within a *q*-range of 0.085–0.11 nm^−1^ for collagen [Figs. 3 ▸(*d*)] and 0.231–0.367 nm^−1^ for myo­fibrils [Fig. 3 ▸(*g*)], asymmetric intensity of scattering (*a*_asym_) corresponding to the anisotropic scattering intensity and the phase shift (θ_s_) denoting the direction of the anisotropic scattering. The degree of orientation was defined as the ratio *a*_asym_/*a*_sym_ [Figs. 3 ▸(*f*) and 3 ▸(i)]. Combined plots of the orientation direction (θ_s_) represented as hue according to the colourwheel and the anisotropic scattering represented by the value [Figs. 3 ▸(*e*) and 3 ▸(*h*)] were generated to visualize the orientation of collagen and myo­fibrils, respectively. Note that the direction of the collagen long axis is along the meridional scattering orientation, showcased using the colourwheel [Fig. 3 ▸(*e*)], while for myo­fibril the direction of the long axis is perpendicular to the equatorial scattering orientation that is exhibited using the colourwheel [Fig. 3 ▸(*h*)].

#### 2.3.3. Analysis of scattering from collagen

In the connective tissue region, in each pixel the opposite angular segments corresponding to the meridional scattering from collagen were identified as the main scattering direction (θ_s_). The corresponding intensity (*I*) *versus* scattering vector (*q*) curves were subjected to background subtraction followed by Gaussian peak fitting over the collagen fibril’s fifth- and sixth-order peaks to estimate the maximum peak position or conversely the real space *D*-period and the full width at half-maximum (FWHM) of the peak or the extent of heterogeneity in the spacing of the collagen fibrils.

The *D*-period of the collagen can be estimated using Bragg’s law,
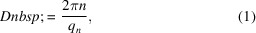
where *n* is the order of the meridional ‘Bragg’ peak used for the calculation and has been estimated using fifth- and sixth-order meridional collagen peaks (*n* = 5, 6). The first peak was excluded from the fitting operation because it had too few data points, while the third peak was also left out due to overlap with muscle peaks.

Dehydration of collagen is apparent through structural changes reflected in the SAXS signal. It has been reported that dehydration of collagen occurs in two stages: the first stage resulting in an increase in the gap-to-overlap ratio (or alternatively, a decrease in the overlap/*D*-period ratio), a decrease in fibril diameter and a decrease in the pitch of the helical tropocollagen molecules and the subsequent stage, resulting in a shrinkage of the *D*-period (Haverkamp *et al.*, 2022 ▸). The overlap/*D*-period ratio (*O*/*D*) was calculated using the equation
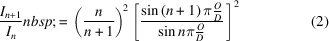
where *I_n_* and *I*_*n*+1_ are the integrated intensities of the consecutive meridional peaks, with *n* and *n*+1 being the consecutive orders of the peaks. In this study, the fifth and sixth peaks have been utilized for the calculation since these peaks were visible in almost all samples. In an undamaged and hydrated collagen I fibril, the ratio of the overlap/gap region has been demonstrated to be 0.46*D*/0.54*D* (Petruska & Hodge, 1964 ▸; Hodge & Schmitt, 1960 ▸). While the above equation is a crude approximation and has multiple solutions, only the structurally reasonable solution was considered (Inamdar *et al.*, 2017 ▸; Xu *et al.*, 2020 ▸).

Dehydration damage to collagen fibrils is alternatively visualized by the ratio of the peak intensity areas of the sixth to the fifth collagen peaks (*I*_6_/*I*_5_), an increase in which has been correlated with dehydration damage to collagen fibrils (Fratzl & Daxer, 1993 ▸; Buchanan *et al.*, 2019 ▸).

#### 2.3.4. Analysis of scattering from myo­fibrils

The equatorial intensity peaks from the myo­fibrils, *I*_1,1_ and *I*_1,0_, were fitted using Gaussian peak fitting, to estimate the corresponding lattice plane distances, *d*_1,1_ and *d*_1,0_, from the peak positions and compare the width of the peaks for different samples. The equatorial scattering direction was extracted in each pixel to define the segments of the intensity curve for the peak fitting. The lattice parameters were calculated using
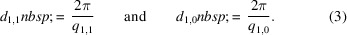
The ratio *I*_1,1_/*I*_1,0_ has been linked to the structural rigidity of the muscle fiber organization. When muscle fibers are at rest, the ratio is low, whereas, in conditions of rigor, there is a many-fold increase in this numerical ratio. This is due to the redistribution of myosin mass from the thick filament towards the thin filament (Huxley, 1968 ▸). While the resting muscle is a feature of living relaxed muscle, rigor muscle is due to a stiffening of the muscle temporarily due to contraction of muscles or permanently after death, a natural process called rigor mortis.

## 3. Results and discussion

### 3.1. Collagen: axial arrangement

**Figure 4:**
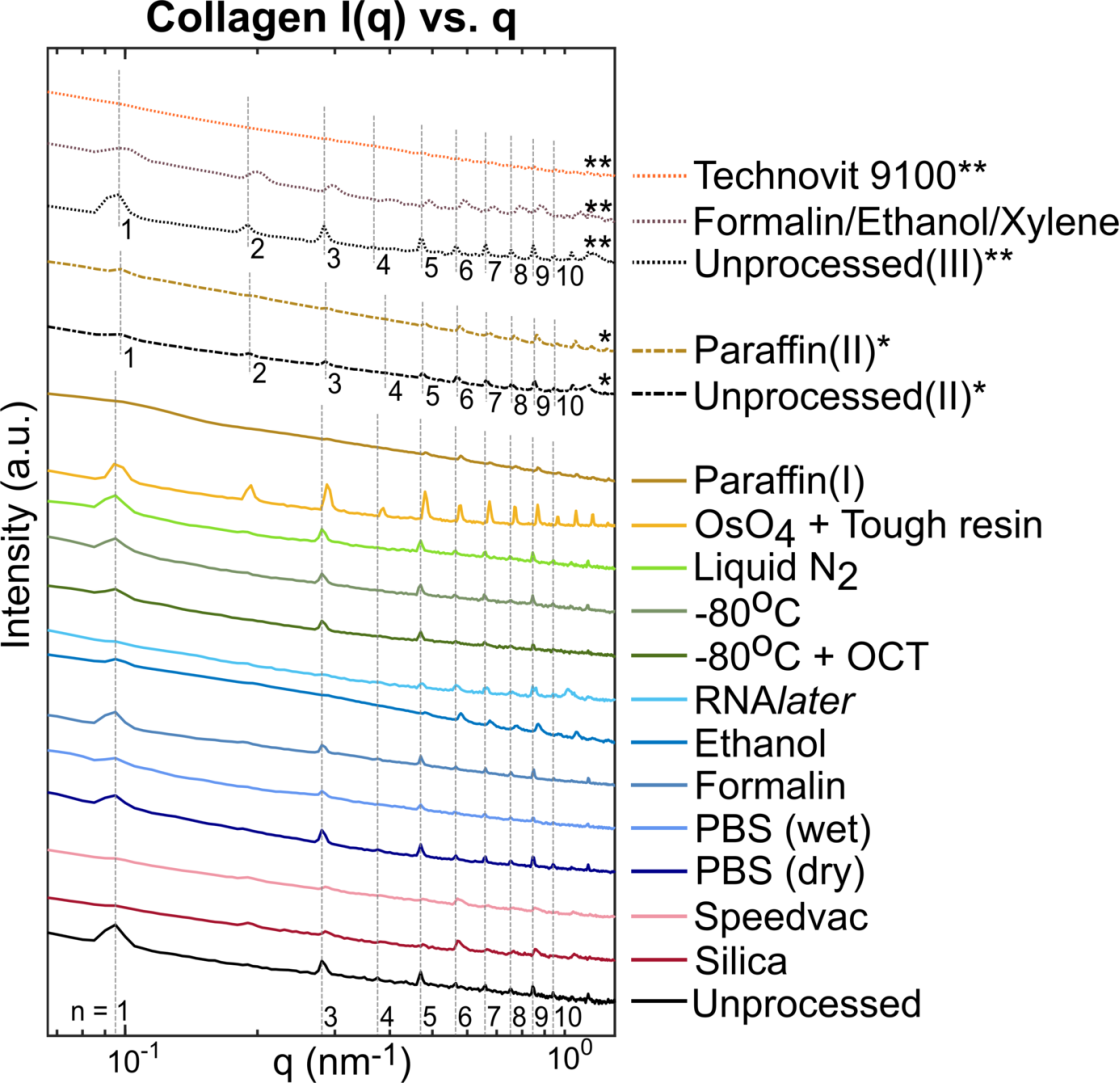
The typical meridional peak intensity *I*(*q*) *versusq* plots obtained from collagen data points in sample measurements. The sample types are mentioned on the right in the same order as the plots. The plots for the paraffin(II)-embedded sample, as well as its reference sample, have been marked with (*) since they were per­formed on a different piece of meat. Similarly, Technovit 9100-embedded sample with its unfixed and fixed references are marked with (**). The vertical dashed lines in gray depict the peaks of order ‘*n*’ of the collagen *D*-spacing for the unprocessed control samples.

Representative 1D scattering distribution plots in the meridional (axial) direction for the samples (Fig. 4 ▸) show that all the samples, except for the Technovit 9100-embedded sample, retained some features of collagen meridional scattering that enabled differentiation between the collagenous and non-collagenous regions. However, the signal varied widely across the different preservation techniques, both in terms of peak positions and relative intensities. The first-order collagen peak was prominent in all preparation techniques except preservation using silica beads, SpeedVac, ethanol and RNA*later*, and embedding in paraffin(I) after prolonged formalin storage. In the first two cases, the peak was only visible in a few scan points with a significantly reduced intensity. This can be attributed to at least a partial dis­integration of the *D*-period repeating structure, possibly due to dehydration induced shrinkage and breakdown of intrafibrillar bonds. The peak was entirely indiscernible in the paraffin(I)-embedded sample with prolonged storage in formalin and was replaced by a very broad shoulder region, while the higher order peaks were also only faintly visible; in contrast, the paraffin(II)-embedded sample with optimized timing of all steps retained all characteristic peaks but with much lower intensity. This illustrated the importance of avoiding prolonged fixation times, since all other variables were unchanged and highlighted that, while paraffin-embedding produced changes, it did not entirely eradicate structural peaks. It remains unclear why Technovit-embedded tissue lost all characteristic peaks. The preceding treatment with formalin, ethanol and xylene was ruled out as the cause of entire structural loss, since a piece of fixed sample from the same source and pre-treatment (Formalin/Ethanol/Xylene in Fig. 4 ▸), except embedding, was found to retain the collagen structure.

**Figure 5:**
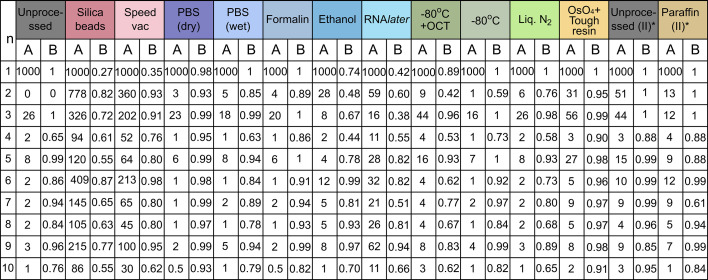
List of normalized intensities of meridional peaks of orders *n* = 1 to 10, and their frequency of occurrence in different processed samples. ‘A’ is the median meridional peak intensity of collagen peaks over the entire sample, normalized against first-order peak intensity, and ‘B’ is the fraction of total collagen data points with a visible peak over the full sample, shown from peak orders *n* = 1 to 10. (*) Statistics for the paraffin(II)-embedded sample should be compared against its own reference, *i.e.* unprocessed(II).

The relative intensity between the different order peaks (Fig. 5 ▸) that depend on the electronic density distribution within the *D*-period, and the frequency of occurrence of peaks, showed a large variation across samples. The second-order collagen peak appeared in all samples in the first set except the unprocessed sample, demonstrating that some degree of structural change occurs with all processes of preservation. Since the latter maintained hydration, the gap and the overlap region are of similar lengths that lead to a damping of the second peak (Giannini *et al.*, 2021 ▸; Terzi *et al.*, 2023 ▸). Note, however, that this is not the case for the second and third set of unprocessed samples, which show that there are inherent structural differences in the assembled collagen fibrils from different sources which is further affected by discrepancies in storage prior to purchase. This is also why having different references was important for different sample sets.

Odd-order peaks were more prominent, while even-order peaks were almost absent in the unprocessed sample. Many fixed samples had a higher incidence of the latter with an amplified intensity and a reduction in prevalence of odd orders. The higher order peaks, especially sixth order and above, were much more intense in many fixed samples, like silica beads, SpeedVac, ethanol and RNA*later*, compared with the unprocessed sample. This was ascribed to the dehydration effect changing the lengths of gap and overlap regions, causing an increase in the electron density contrast between the regions. The remaining even order peaks, as well as the higher order peaks, were amplified in these samples as well, when compared with the rest, indicating more structural differences within the *D*-period due to the removal of water molecules. This is consistent with earlier studies of collagen dehydration that was shown to induce tensile stress in the fibrils due to osmotic changes (Masic *et al.*, 2015 ▸; Bertinetti *et al.*, 2015 ▸). Note that the relatively high intensities of all peaks in these four samples (Fig. 5 ▸) is due to the low intensity of the first-order peak, which has been used as a normalization factor for other peaks. The cryopreserved samples in general retained the high intensity odd-order peaks and less-intense even-order pattern; however, the OCT-embedded sample showed a noisier intensity distribution than the rest. This was probably due to large scatterers present in OCT, like polyethyl­ene glycol (PEG) and polyvinyl alcohol (PVA), which are water soluble and can be washed away prior to measurement. The Os-stained and tough-resin-embedded sample showed high peak intensities across all orders, caused by a larger scattering contrast from the heavy osmium ions directionally bound to collagen.

In contrast to the unprocessed sample in the first set, the unprocessed(II) sample, which had a different source, showed a higher prevalence of more intense even-order peak intensities, which can be attributed to a slightly different collagen architecture. The paraffin(II) sample showed a similar structural pattern, except for lowered peak intensities for low-order peaks and a more intense sixth peak compared to its fifth peak.

In a nutshell, the PBS-, formalin- and to a lesser extent the cryopreserved samples maintained the closest resemblance to the unprocessed sample in this respect.

### 3.2. Collagen: peak analysis

**Figure 6:**
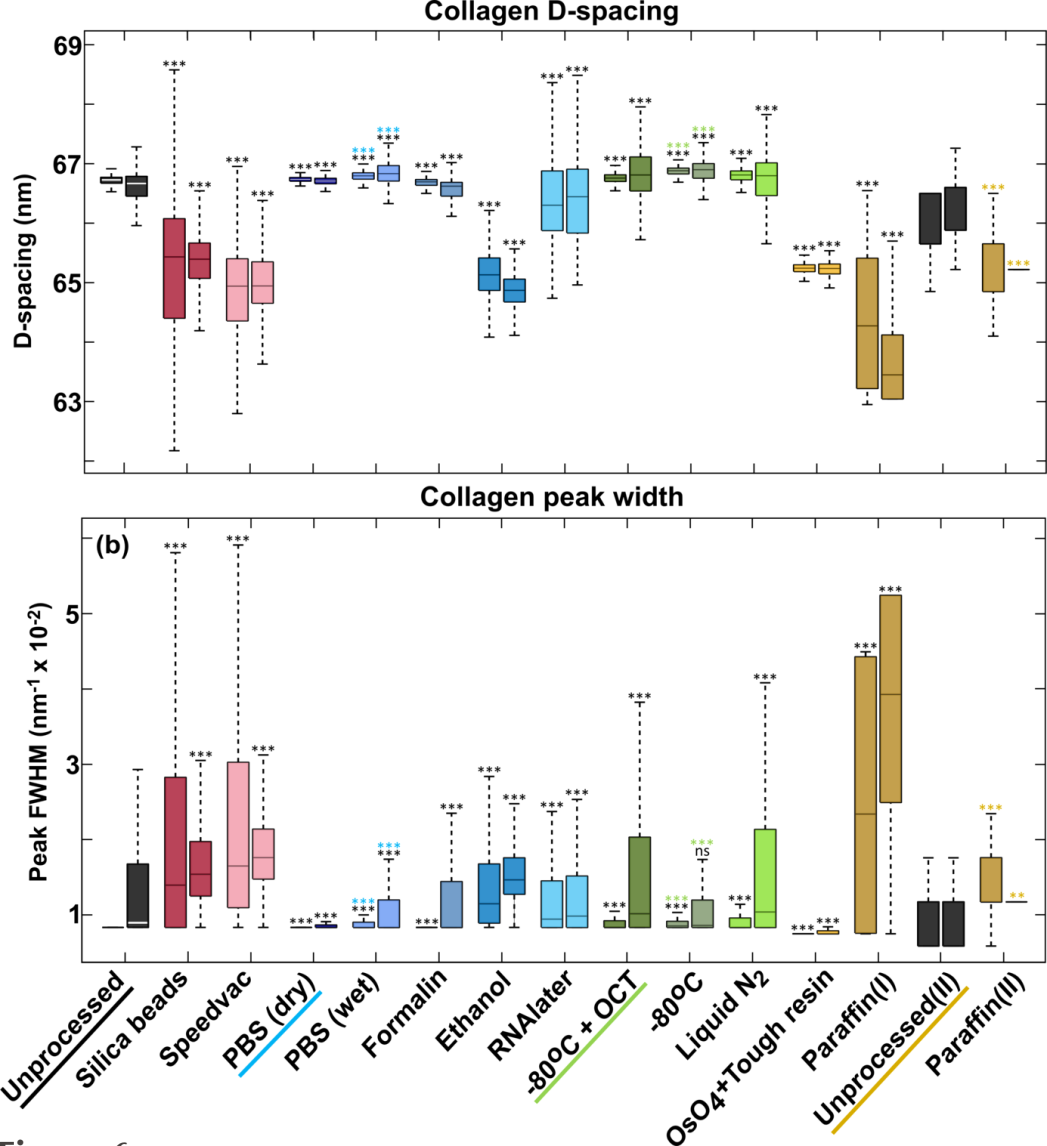
(*a*) The distribution over pixels in the samples of the collagen *D*-period calculated from fifth- and sixth-order peak positions, for non-zero meridional intensity collagen scan points in all samples, is shown using box plots. Individual sample labels along the *x* axis contain two box plots each, with the left one corresponding to the *D*-period calculated from the fifth peak and the right plot calculated from the sixth peak. (*b*) The corresponding FWHM distribution of the fifth (left) and sixth (right) peaks are displayed in the same order. Statistical significance was tested against the underlined samples (colour-coded) using Wilcoxon signed-rank test (‘ns’/not significant → *P* > 0.05; ‘*’ → *P* ≤ 0.05; ‘**’ → *P* ≤ 0.01; ‘***’ → *P* ≤ 0.001). Parameters of samples from silica beads to paraffin(I) were statistically tested against the unprocessed sample; PBS (wet) is additionally tested against PBS (dry), while the −80°C-preserved sample is tested against a −80°C+OCT-preserved sample. The paraffin(II)-embedded sample was statistically tested against the unprocessed(II) sample. The Technovit 9100-embedded sample was excluded from this analysis due to non-existent collagen peaks.

The fifth- and sixth-order peaks were chosen for sample-wide analysis because they appeared in all samples, except for the Technovit-embedded sample, and could be used for statistical comparison between samples. The peaks displayed large variation within each sample, so for the statistical comparison we investigated the distribution of peak parameters over the pixels in each sample. The *D*-period and the width were calculated from both the peaks, and the sample-wide distributions were visualized as box plots [Figs. 6 ▸(*a*)–(*b*)].

Using the fifth-order peak position, the median collagen *D*-period in the unprocessed sample was found to be 66.727 nm. Samples preserved in PBS (dry and wet) and formalin, and cryopreserved samples had a *D*-period median very close to it, with a deviation of less than 0.25% and a similar distribution range. This indicates that the fibrillar arrangement of collagen remained intact in these samples. On the other hand, samples preserved using silica beads, SpeedVac, ethanol and paraffin(I) embedding showed a reduction in the median *D*-period by 2% or more when compared to the unprocessed reference. They also had a large distribution range across each sample, which can at least be partially attributed to a low signal-to-noise ratio due to low-intensity fifth-order peaks. This is in addition to much fewer collagen points retaining the fifth peak (see ‘B’ in Fig. 5 ▸), indicating at least a partial loss of structural integrity. The RNA*later*-processed sample showed a relatively closer median to the unprocessed sample, but also had a large sample-wide variation in measurements. The tough-resin-embedded sample showed the highest deviation, with a much lower median *D*-period (3.64% shrinkage), which was possibly a result of dehydration caused by pre-embedding steps, as well as shrinkage due to solidification of the ep­oxy resin. However, the narrow distribution across the sample and the retention of the collagen peaks by almost all data points indicate that the structure framework remained intact. As a general trend, the sixth-order peak was highly pronounced in samples which had a damped fifth (along with first and third) peak and *vice versa*. This led to a wider distribution range in *D*-period calculated using the sixth peak, which was especially pronounced in the cryopreserved samples. The high-intensity sixth-order peaks in the silica-dried, SpeedVac and ethanol-preserved samples led to a higher signal-to-noise ratio over this peak which was likely responsible for the narrower *D*-period distribution.

The same pattern was observed for the peak-width distribution: samples with a more prominent fifth peak had a narrower fifth-order peak width, while those with a prominent sixth peak showed a narrower sixth-order peak width. Note that the lower limit being consistent in the width distribution across samples is due to the resolution limit caused by *q*-binning in the radial direction; any values lower than this limit is therefore indeterminate. The fifth peak width remained low with negligible variation for the PBS (dry) and formalin-fixed samples, along with the unprocessed samples. The PBS-immersed (or wet) sample and cryopreserved samples were also observed to have a low yet more discernible distribution in width for both. Silica beads-, SpeedVac- and RNA*later*-fixed samples had a higher median value for the fifth peak width, along with greater variation in distribution, indicating a greater degree of disorder in the collagen organization. The tough resin-embedded sample had a very narrow peak-width distribution for both peaks. The peak width here is biased due to the increase in intensity caused by Os staining. The paraffin(I)-embedded sample had a very wide peak-width distribution due to much less discernible collagen peaks, resulting in a low signal-to-noise ratio for both peaks.

The paraffin(II)-embedded sample had a lower *D*-period compared to unprocessed(II) sample, with the median change around 2%. Since the sixth peak was more prominent in paraffin(II), the distribution range for the sixth peak position and width was very narrow compared to unprocessed(II), while the trend was similar for other peaks, except for the first peak width exhibiting the opposite trend.

In essence, preservation in PBS and formalin, and cryopreserved samples had the closest resemblance to the unprocessed sample.

### 3.3. Collagen: *I*_6_/*I*_5_ ratio and overlap/*D*-period ratio

**Figure 7:**
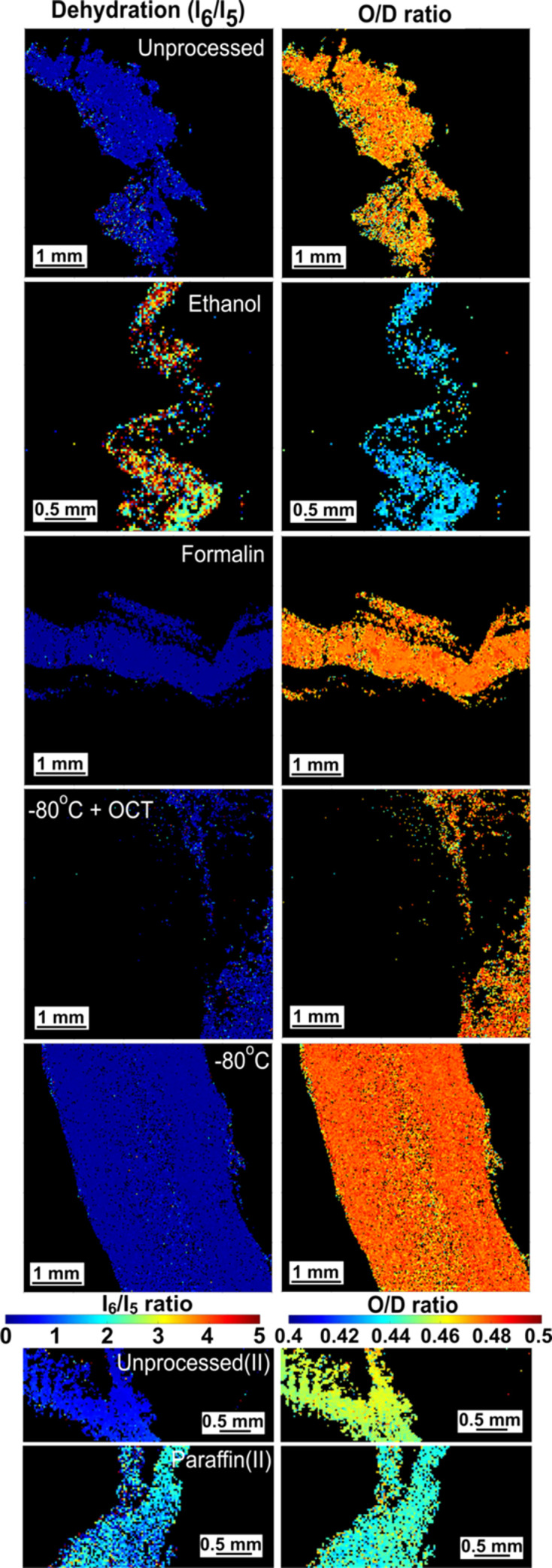
*I*_6_/*I*_5_ ratio (left) and *O*/*D* ratio (right) are computed in commonly used sample-processing techniques. Other samples are shown in Fig. S2 of the supporting information.

The extent of dehydration was compared using the ratio*I*_6_/*I*_5_ (Fratzl & Daxer, 1993 ▸; Buchanan *et al.*, 2019 ▸; Basil-Jones *et al.*, 2010 ▸), as well as a change in overlap to *D*-period (*O*/*D*) ratio. The area plots corresponding to the most commonly used preservation techniques are shown in Fig. 7 ▸, while the rest have been included in Fig. S3 of the supporting information.

The samples fixed using silica beads, SpeedVac, ethanol and, to a lesser extent, RNA*later*, paraffin(I)- and paraffin(II)embedded samples showed a higher *I*_6_/*I*_5_ ratio compared to the rest of the samples. This same set of samples showed a decrease in *D*-period in the previous section.

A high degree of dehydration is due to a loss of hydrogen bonds with water molecules that leads to a decrease in the overlap region of the *D*-period (Haverkamp *et al.*, 2022 ▸; Masic *et al.*, 2015 ▸). The *O*/*D* ratio in the unprocessed sample was found to be in the range 0.46–0.48, aligning with the literature (Petruska & Hodge, 1964 ▸; Hodge & Schmitt, 1960 ▸). The parameter had a similar value in the formalin, PBS, cryopreserved and ep­oxy-embedded samples. Similar to the *I*_6_/*I*_5_ ratio, the silica beads-, SpeedVac-, ethanol- and RNA*later*-preserved samples showed a high disparity from the unprocessed sample, with values ranging between 0.42 and 0.44. The paraffin(I)-embedded sample had an *O*/*D* ratio in the range 0.43–0.44 and the paraffin(II)-embedded sample also lowered this ratio to 0.43–0.44, compared to the range of 0.45–0.46 in the unprocessed(II) sample.

The two parameters – dehydration and *O*/*D* ratio – are linked and thus show a similar trend across samples. Note that, while a change in *O*/*D* ratio is often an indication of dehydration, it can also be caused by a change in nanostructure due to cross-linking or the application of mechanical stress (Kamml *et al.*, 2023 ▸; Sasaki & Odajima, 1996 ▸).

### 3.4. Collagen: orientation analysis

**Figure 8:**
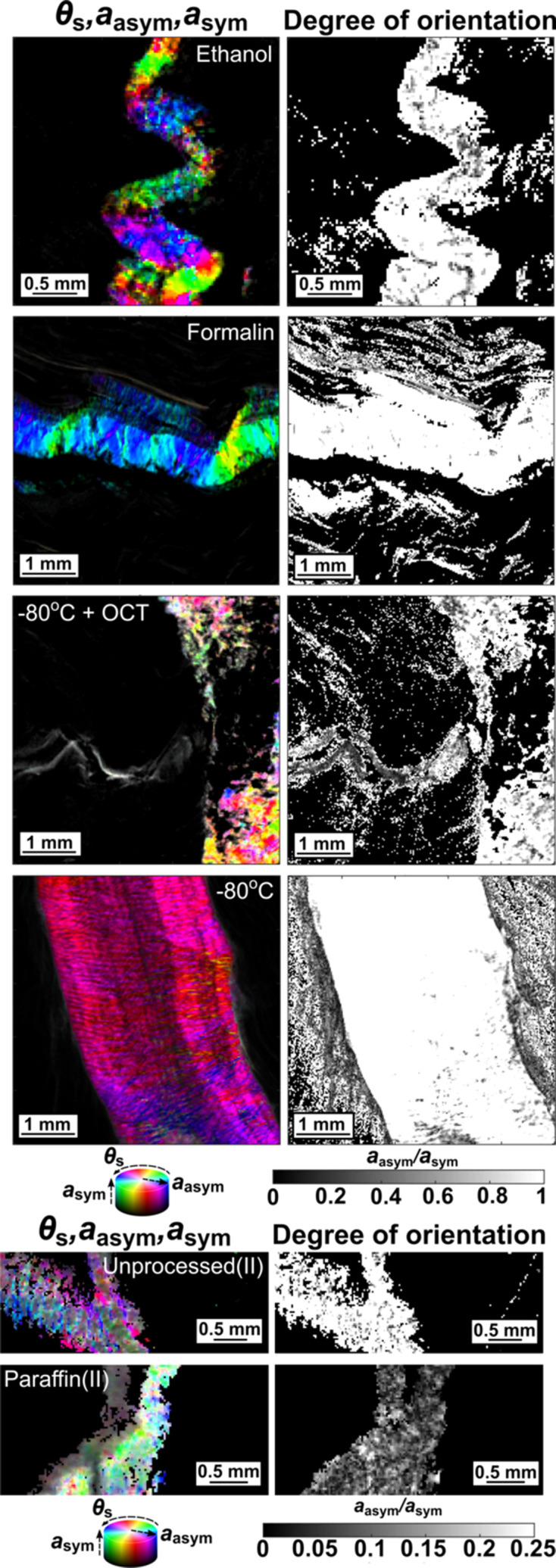
Scanning SAXS images of various samples over the *q*-range = 0.085–0.11 nm^−1^. On the left, images are plotted using an HSV scale, where the hue scales with the orientation of scattering (θ_s_), according to the colourwheel displayed, the saturation scales with the asymmetric amplitude of scattering (*a*_asym_) and the value scales with the symmetric scattering amplitude (*a*_sym_). On the right, the images illustrate the degree of orientation (*a*_asym_/*a*_sym_), that scales from 0 to 1 for all figures, except for the unprocessed(II)- and paraffin(II)-embedded figures, where it scales from 0 to 0.25. Other samples are shown in Fig. S3 of the supporting information.

The collagen region in all the samples showed asymmetric scattering over the first-order peak region, which made it possible to distinguish these regions and assess the average orientation of the collagen fibrils at different scan points (Fig. 8 ▸ and Fig. S4 of the supporting information). However, the silica beads-, SpeedVac- and RNA*later*-preserved samples did not retain the compact collagen organization seen in the other samples, but showed a sparser distribution of collagen and not a well-defined degree of alignment, which probably means that the structural integrity of the connective tissue region is lost. The ethanol-processed sample retained a compact collagen alignment but showed severe wrinkling due to dehydration-induced structural changes. The degree of orientation shown by the collagen in the paraffin(II)-embedded sample was lower than its reference, which could be a result of a smaller first collagen peak in the former. However, this could have been caused by a difference in the thickness of the collagen layer between the samples. The unprocessed(II) sample itself showed a lower degree of orientation compared to the differently sourced first set of samples.

### 3.5. Myofibril: peak analysis

**Figure 9:**
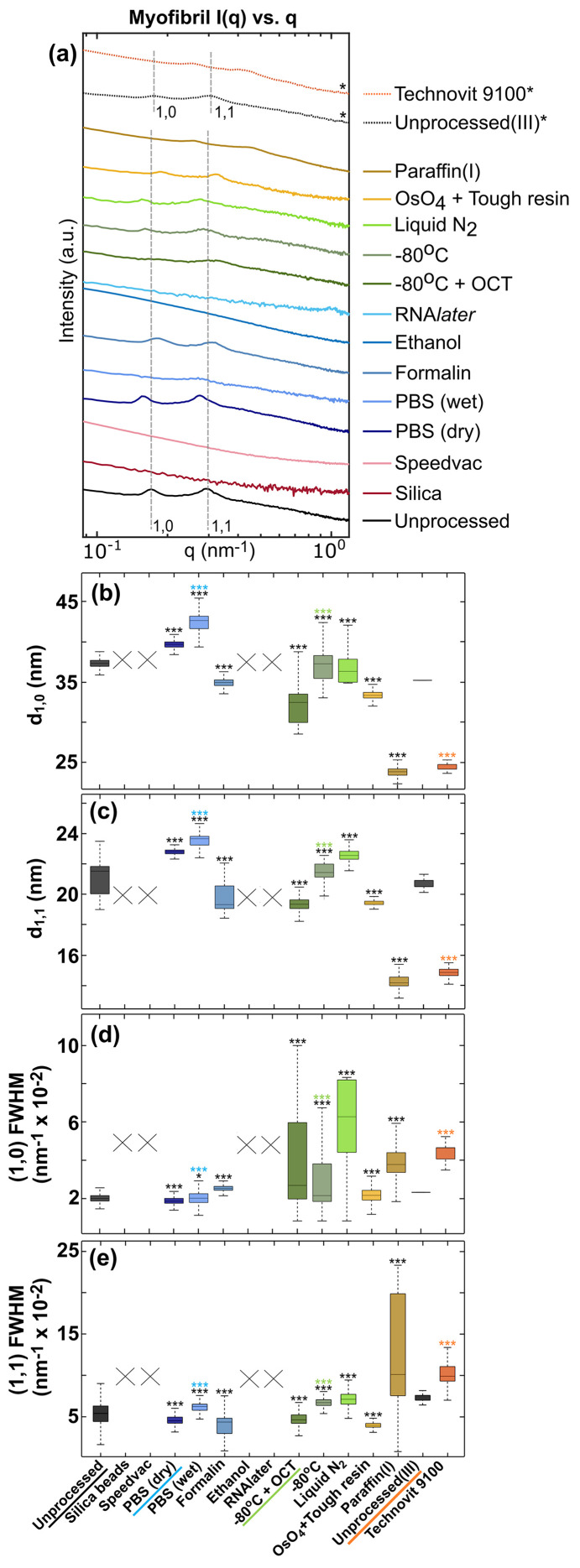
Analysis of muscle equatorial scattering. (*a*) The typical equatorial scattering intensity *I*(*q*) *versusq* plot obtained for all samples. The dashed vertical lines in gray show the *d*_1,0_ and *d*_1,1_ peaks for the unprocessed control sample. The sample types are mentioned on the right in the same order as the plots. Plots are shown for the Technovit 9100-embedded sample and its reference sample, unprocessed(III), with the asterisk (*) corresponding to a different sample source. (*b*, *c*) Box plot distributions of muscle equatorial peaks, *d*_1,0_ and *d*_1,1_, respectively, from the segmented muscle region in samples. (*d*, *e*) The corresponding peak-width (FWHM) distributions. Statistical significance was tested against the underlined samples (colour-coded, similar to Fig. 6 ▸) using Wilcoxon signed-rank test (‘ns’/not significant → *P* > 0.05; ‘*’ → *P* ≤ 0.05; ‘**’ → *P* ≤ 0.01; ‘***’ → *P* ≤ 0.001).

We focused on the analysis of the more prominent equatorial peaks, *I*_1,0_ and *I*_1,1_, arising from the cross-sectional lattice arrangement, rather than the meridional peaks which were only very faintly visible in a few samples, probably caused by the dense tissue environment and the influence of the cutting direction. The scattered intensity from myo­fibrils [Fig. 9 ▸(*a*)], in general, were one order of magnitude lower than that of collagen. The fixation and embedding methods also had a more drastic effect on the structure of the muscle fibers since these tissues are very sensitive to the osmotic properties of the surrounding media (Bozler, 1965 ▸; Ruiz & Souza, 2008 ▸), which needs to be considered during structural studies of muscles. Fixations including silica beads, SpeedVac, ethanol and RNA*later* caused the disappearance of the double equatorial peaks entirely, indicating that the lattice structure was either entirely destroyed or drastically and ununiformly distorted while the other samples retained the characteristic features at least to some extent, at least partially preserving structural rigidity. The cryopreserved, as well as the tough-resin embedded, samples showed a noisy signal due to lower scattering intensity. In the case of cryopreservation, this indicates that the effect of ice-crystal formation and subsequent thawing does affect the muscle structure. The scattering from the unprocessed(II) sample lacked any features of myo­fibril to begin with and hence the paraffin(I)-embedded sample had to be used for analysis instead of paraffin(II). In contrast to collagen, where the peaks disappeared, the Technovit 9100-embedded sample retained the equatorial myo­fibril features, that has been compared to its reference.

The distribution of equatorial peak positions in terms of their *d*-spacings, *d*_1,0_ [Fig. 9 ▸(*b*)] and *d*_1,1_ [Fig. 9 ▸(*c*)], showed that formalin and PBS (dry) most closely resembled the muscle structure in the unprocessed sample, with a small change in lattice parameters. This was because the volume of muscle fibers has been shown to shrink in formalin (Leonard *et al.*, 2022 ▸), causing a small shortening of the lattice parameters, while PBS slightly increases the fiber volume due to a hydration effect (Ward & Lieber, 2005 ▸), leading to longer lattice parameters. Both paraffin(I) embedding and Technovit 9100 embedding caused a notable shift (over 30%) in equatorial *d*-spacing. In the paraffin(I) sample, the medians shifted more than 30% in comparison with the unprocessed sample, which is consistent with previous studies on muscles (Nielsen *et al.*, 1995 ▸). Similarly, the Technovit 9100 embedding lowered *d*_1,0_ and *d*_1,1_ by 31 and 29%, respectively, in comparison with unprocessed(III). The tough-resin-embedded samples also showed a shortening of the lattice parameters, although to a much lower extent than for paraffin(I) and Technovit 9100 embedding. There was also a substantial lowering of intensities in the muscle peaks of the paraffin(I)-embedded, wet PBS and OCT-embedded samples.

Formalin and PBS-preserved samples showed a comparable peak width to unprocessed sample for both peaks [Fig. 9 ▸(*d*) and 9 ▸(*e*)]. The cryopreserved samples had a noisier distribution over the (1,0) peak range than the (1,1) peak, causing a large variation in the (1,0) peak-width distribution. This could indicate that the ice-crystal damage disproportionately changes in structure along the (1,0) lattice plane when compared to the (1,1) plane. Paraffin(I), on the other hand, showed a higher (1,1) peak-width distribution range, while the Technovit 9100-embedded sample showed a higher peak width than unprocessed(III) for both peaks.

### 3.6. Myofibril: ratio of equatorial peak intensities

**Figure 10:**
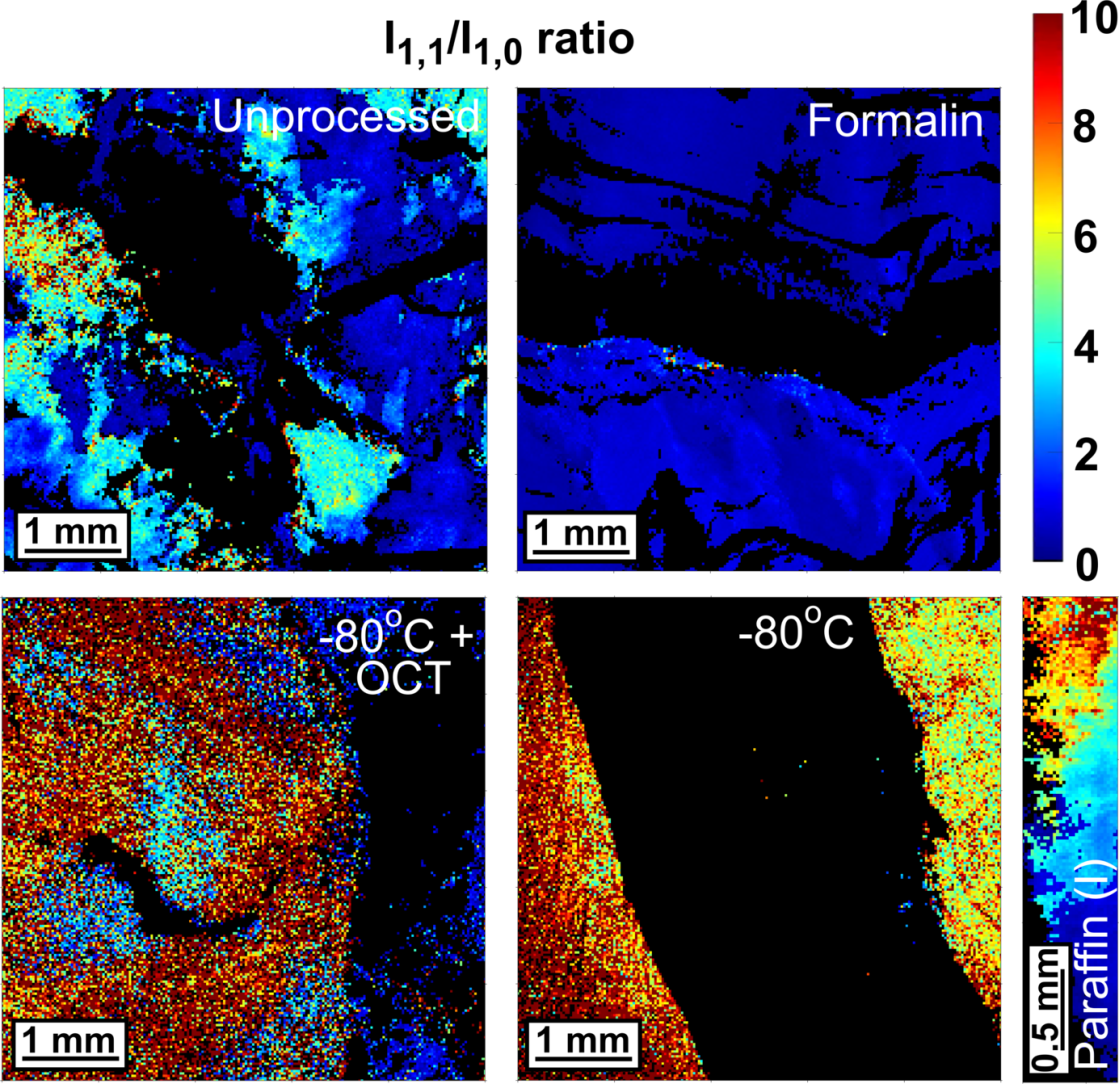
Image plots depicting the *I*_1,1_/*I*_1,0_ ratio in common tissue-processing types. The rest of the samples are included in Fig. S4 of the supporting information.

Comparing the ratio of the equatorial peak intensities showed significant variation among the samples (Fig. 10 ▸ and Fig. S5 of the supporting information). Interestingly, the unprocessed sample showed a disparity in this ratio with some regions showing a higher ratio than others. However, this is not entirely unexpected since the sample was obtained and stored for 72 h pre-measurement which might have caused uneven hydration over the sample area, as well as the onset of rigor, both of which would have led to a change in the lattice parameters (Liu *et al.*, 2018 ▸). This inconsistent pattern was also shown by the paraffin(I)-embedded sample, which also exhibited a shortened *d*-spacing. Formalin led to a stabilization of the muscle structure by forming cross-links between muscle proteins like myosin and actin (Kiernan, 2000 ▸), in a fashion similar to how it stabilizes collagen, and thus maintained a low and almost uniform ratio throughout. Technovit 9100 embedding also preserved the *I*_1,1_/*I*_1,0_ ratio, even though it significantly shortened the lattice parameters. Both PBS (dry and wet) samples had a moderately elevated ratio with some heterogeneity across the samples. However, the cryopreserved and tough-resin-embedded samples had a very high ratio, indicating significant structural change. In the case of cryopreservation, the elevated *I*_1,1_/*I*_1,0_ ratio can be due to structural damage caused by ice-crystal formation (He *et al.*, 2020 ▸) or due to thaw rigor-induced shortening (Xiong & Blanchard, 1993 ▸).

### 3.7. Effect of sample thickness

**Figure 11:**
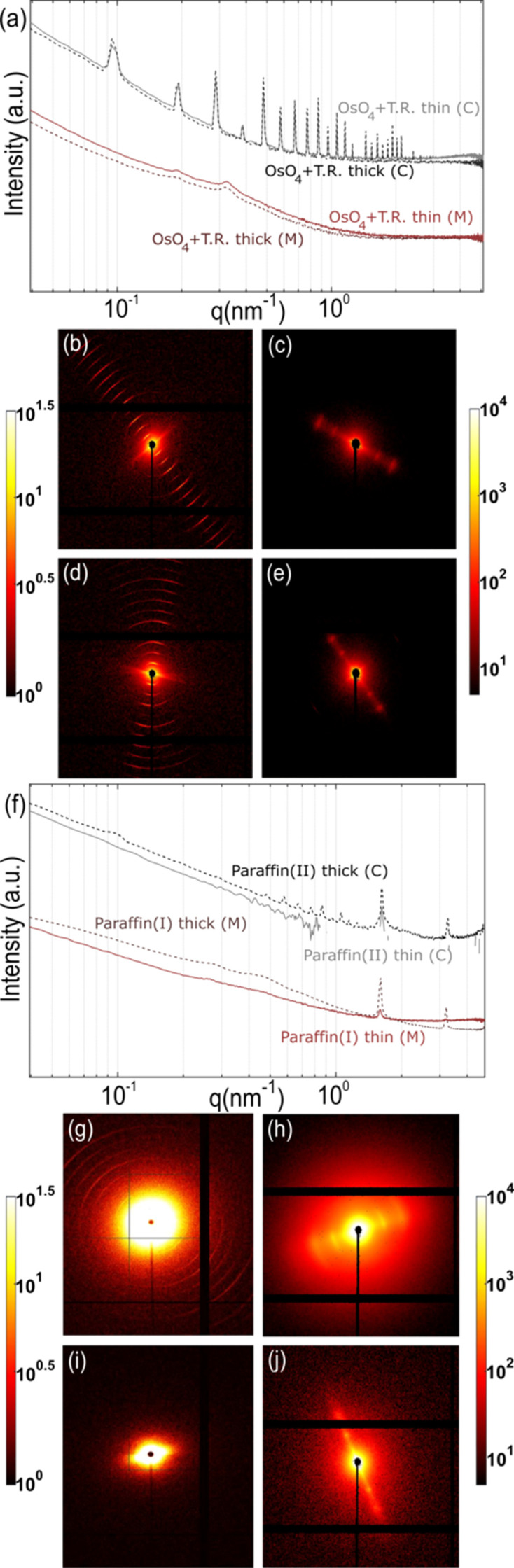
(*a*) 1D scattering intensity *versusq* plots shown in tough-resin (TR) thick (1 mm) and thin (300 µm) samples for collagen (C) and muscle (M) regions; SAXS pattern for (*b*) collagen and (*c*) muscle in tough-resin thick sample; and SAXS pattern for (*d*) collagen and (*e*) muscle in thin sample. (*f*) 1D scattering intensity *versusq* plots shown in paraffin-embedded thick (1 mm) and thin (30 µm) samples for collagen (C) and muscle (M) regions; SAXS pattern for (*g*) collagen and (*h*) muscle in thick paraffin(II)- and paraffin(I)-embedded samples, respectively; and SAXS pattern of (*i*) collagen and (*j*) muscle in the corresponding paraffin-embedded thin samples.

To estimate the effect of sample thickness on SAXS measurements in soft tissues, two different thicknesses were investigated for tough-resin- [Figs. 11 ▸(*a*)–11(*e*)] and paraffin-embedded samples [Figs. 11 ▸ (*f*)–11(*j*)] with the same exposure time of 100 ms. The 1D scattering plots of the former 1 mm-thick sample and 300 µm-thin sample [Fig. 11 ▸(*a*)] did not show any major differences in the peak characteristics in both the collagen (C) and the muscle (M) regions, even though the average symmetric scattering was higher in the thick sample (not depicted here). The typical SAXS pattern for collagen and muscle were also indistinguishable for both thicknesses. It is, however, important to remember that treatment with OsO_4_ leads to amplified scattering which might alleviate the need for having very thick samples for scattering experiments in tough-resin-embedded samples, but it skews the intensity ratios of the *D*-period peaks in the process.

In contrast, the paraffin-embedded samples, which had higher contrasting thicknesses, *i.e.* 1 mm thick *versus* 30 µm thin, were more dissimilar. Note that the collagen regions were compared in paraffin(II) embedding while muscle regions were compared in paraffin(I) embedding, due to the lack of muscle peaks observed in paraffin(II) and its reference. The scattering signal in the collagen (C) region of the thin sample was extremely noisy and incomplete since the sample was not thick enough to generate enough scattering signal for the 100 ms exposure time used. The muscle (M) peaks were visible but significantly dimmed.

It is therefore important to consider that having thin samples, even if they contain highly organized structures within, can lead to a scattering signal below the detection limit due to too few scatterers, especially if the method of preservation already leads to the dampening of these features. Conversely, if the samples are too thick, we might miss spatial resolution and therewith some features since the SAXS signal is averaged across the sample’s thickness. In addition, there are practical considerations associated with the sectioning of thin samples with different methods.

In principle, the signal-to-noise ratio in thinner samples can be increased by using a higher exposure time. In the first beamtime, as an example, the radiation dose accumulated is calculated according to Yu *et al.* (2025 ▸), assuming a mass attenuation coefficient for soft tissue, μ/ρ_m_, of 2.6 cm^2^ g^−1^ (Seltzer, 1995 ▸), resulting in 10 kGy, which is significantly lower than the limit reported in the literature for soft tissues (Fernández *et al.*, 2002 ▸; Barreto *et al.*, 2023 ▸). However, this limit depends on many factors, such as level of hydration (Le Caër, 2011 ▸) and sample thickness (Yu *et al.*, 2025 ▸; Wu *et al.*, 2023 ▸), and therefore a radiation-damage check is mandatory at the beginning of the experiment. Another downside of longer exposure times is an increase in the overall measurement time.

## 4. Limitations

Due to logistical issues, three different sample sets from three different sources were used to complete the study, along with four separate beam-time measurements. Although a separate reference has been used for each sample set for the purpose of comparison, different sources can lead to different results even when all other experimental parameters are kept the same. There is also the possibility of sample-to-sample differences even when they originate from the same source. Unfortunately, only one sample per preparation method could be measured due to time constraints and, therefore, inter-sample variability remains unaccounted for.

In addition to sample-processing methods, other potential causes of sample degradation exist, arising either from the sample environment itself or from interactions between X-rays and the preservation source. Due to the convenience store procurement of the bovine meat, a temporal delay occurred between the slaughter of the source animal and the preparation of the samples, which can be a potential cause of structural alterations. However, this effect can mostly be neglected as the control sample used was obtained from the same source. The cryo-samples were not kept frozen during measurement due to beamline logistics. Since some samples, such as the references and PBS-immersed samples, remained hydrated, there is an additional interaction between the X-rays and the water molecules that generate radicals and increase sensitivity of structural damage to the soft tissues (Le Caër, 2011 ▸). Similarly, tissue stained with heavy metals like osmium increase the absorbed radiation dose that increase sensitivity to radiation-induced structural damage (Ströh *et al.*, 2022 ▸; Paithankar & Garman, 2010 ▸).

The samples were sectioned using a scalpel which could have resulted in some inconsistencies in intra- and inter-sample thickness. The relatively high sample thickness (around 1 mm) implies that there could be changing alignment and other feature parameters within the sample thickness that is averaged out in a SAXS measurement. Some promising techniques, like high-pressure freezing and lyophilization for preservation, could not be per­formed for logistical reasons. The study of the effect of tissue thicknesses has not been extended to commonly used techniques yet, like OCT-embedded ultra-low-temperature freezing. In essence, we consider this to be a starting point in the creation of a catalog listing the effect of fixation/embedding protocols on soft tissues, which can be further built upon with time. With growing interest in X-ray studies of soft tissues, it could be vital to have access to a comprehensive guide to different protocols.

## 5. Conclusion

There is not a single sample-preparation method that would satisfy every requirement and thus the method chosen must be experimental goal-specific and feature-focused. In the case of a collagen study, processing methods like formalin, PBS, cryopreservation and paraffin(II) embedding worked well in preserving the structure. OCT embedding caused some noisy scattering which can be avoided by washing the sample with water before measurement. However, if the distribution of collagen is sparse in the sample and the goal is to look at the alignment and organization of collagen fibers in a thin sample section, Os staining with tough-resin embedding is a good technique because OsO_4_ staining can amplify the collagen signals in the sample, albeit with changed intensity ratios. The investigation of muscle structure, on the other hand, showed that only formalin and PBS preserved the structure closely and cryopreservation did not work well. Four methods of preservation – silica beads, SpeedVac, ethanol and RNA*later* – per­formed dismally in preserving both collagen and muscle structure, while all embedding methods caused at least some amount of shrinkage (lower *d*-spacing) in both structures.

**Figure 12:**
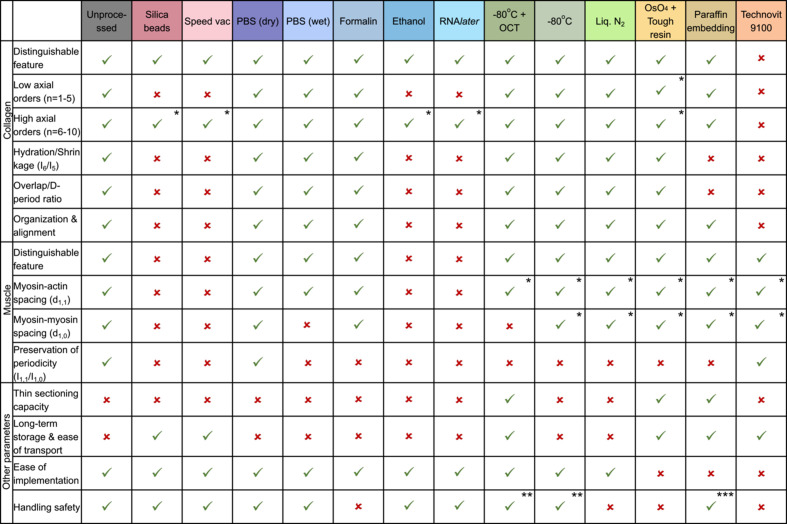
Summary table showing the effectiveness of different sample-preservation methods depending on the parameters to be considered for the experiment. Notes: (*) features are visible but show significant differences from the unprocessed sample; (**) generally safe to handle but snap freezing requires handling with liquid nitro­gen/dry ice; (***) safe to handle post embedding, but often involves formalin fixation as a pre-embedding step.

There are, however, other considerations that should be taken into account before making a decision on the appropriate processing method, such as, the desired thickness of samples which may need embedding or cryosectioning, and the duration of storage needed. The different methods that were probed are listed with some such common criteria to be considered (Fig. 12 ▸), which can aid in planning sample preparation.

## Supplementary Material

sSAXS beamtime setup parameters and additional figures. DOI: 10.1107/S1600577526001530/bon5001sup1.pdf

## Figures and Tables

**Table 1:** List of soft tissue preparation techniques used in different X-ray experiments (list not limited to X-ray scattering)

Soft tissue type	Preservation technique
Muscular tissue	50% (*v*/*v*) glycerol solution at −20°C (Caremani *et al.*, 2021 ▸)*, 10% formalin fixation (Ma *et al.*, 2020 ▸)*, 10% formalin fixation and storage in PBS with 0.04% NaN_3_ (Nicolas *et al.*, 2020 ▸), storage at −160°C with sucrose as cryoprotectant (Ochala *et al.*, 2010 ▸)*.
Adipose tissue (fat)	Storage in liquid N_2_ (Changizi *et al.*, 2005 ▸), storage at −80°C (Sidhu *et al.*, 2011 ▸), 10% formalin fixation (Arboleda *et al.*, 2019 ▸), lyophilization (Conceição *et al.*, 2020 ▸).
Fibrous connective tissue	Storage at −80°C (Sidhu *et al.*, 2011 ▸), 10% formalin fixation (Arboleda *et al.*, 2019 ▸), lyophilization (Conceição *et al.*, 2020 ▸), air drying (De Caro *et al.*, 2013 ▸), PBS with 1 m*M* NaN_3_ and subsequent freezing (Tadimalla *et al.*, 2017 ▸), formalin-fixed paraffin embedding and subsequent dewaxing (Mohd Sobri *et al.*, 2020 ▸).
Nervous tissue	4% paraformaldehyde (PFA) fixed and stored at 4°C in PBS (Georgiadis *et al.*, 2020 ▸), formalin stored for several days/weeks (De Felici *et al.*, 2008 ▸; Jensen *et al.*, 2011 ▸), fixation in 2% PFA–2.5% glutaraldehyde (Inouye *et al.*, 2014 ▸), 1% glutaraldehyde fixation, OsO_4_ staining and ep­oxy-based (tough**) resin embedding (Bosch *et al.*, 2023 ▸).

Notes: (*) samples are muscle bundles permeabilized using skinning solutions; (**) further explained in Section 2.

## Data Availability

Raw data available upon request.
