# Supplementary material for: Effect of soft tissue sample preparation techniques for scanning small-angle X-ray scattering experiments
Source: J Synchrotron Radiat. 2026 Apr 14;33(Pt 3):632–48. doi: 10.1107/S1600577526001530 (PMC13148613; doi:10.1107/S1600577526001530)
Supplement: Supplementary file 1 [file s-33-00632-sup1.pdf]

JOURNAL OF
SYNCHROTRON
RADIATION

Volume 33 (2026)

Supporting information for article:

Effect of soft tissue sample preparation techniques for Scanning Small-Angle X-ray scattering experiments

Atreyee Acharya, Arthur Baroni, Irene Rodriguez-Fernandez, Mads Carlsen and Marianne Liebi

S1. sSAXS beamtime setup parameters

Table S1 List of experimental setup parameters and respective measured samples during the four beamtime experiments

Experimental setup parameters	Beamtime 1	Beamtime 2	Beamtime 3	Beamtime 4
Energy (keV)	12.4	11.2	12.4	12.4
Beam size (µm)	26 x 16	28 x 15	25 x 14	25 x 16
Flux (photons/s)	7.8×10^{10}	1.38×10^{11}	1.04×10^{11}	1.02×10^{11}
Detector	Pilatus 2M	Pilatus 2M	Eiger 9M	Eiger 9M
Sample-detector distance (m)	2.161	2.172	2.120	2.128
q-range (nm^{-1})	0.0050 – 6.3297	0.0045 – 5.3506	0.0040 – 5.8551	0.0043 – 5.8334
Step size (µm)	25 x 25	25 x 25	25 x 25	25 x 25
Samples with their scanned area sizes (mm)	Unprocessed: 5 x 5<br>Silica beads: 5 x 5<br>Speedvac: 5 x 5<br>PBS (dry): 5 x 5<br>PBS (wet): 5 x 5<br>Formalin: 5 x 5<br>Ethanol: 3 x 3<br>RNA*later*: 5 x 5<br>-80ºC+OCT: 5 x 5<br>-80ºC: 5 x 5<br>Liquid N_2: 5 x 5	OsO4+Tough resin: approx. 2 x 1<br>Paraffin(I): 0.6 x 3	Unprocessed (II): 1.5 x 4<br>Paraffin(II): 1.7 x 3.5	Unprocessed(III): 1.25 x 4<br>Technovit 9100: 1 x 5

S2. Segmented regions: Intensity vs. scattering vector, q

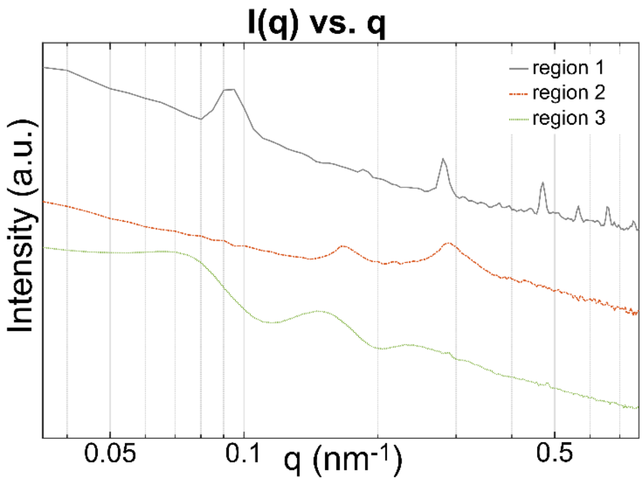

Figure S1 The typical scattering intensity *I(q)* vs scattering vector, *q*, plots corresponding to the three regions in figure 3(c) in main text. Colour codes have been kept the same. (region 1 = collagen; region 2 = muscle; region 3 = unknown)

S3. Collagen: I_6/I_5 ratio and overlap/*D*-period ratio

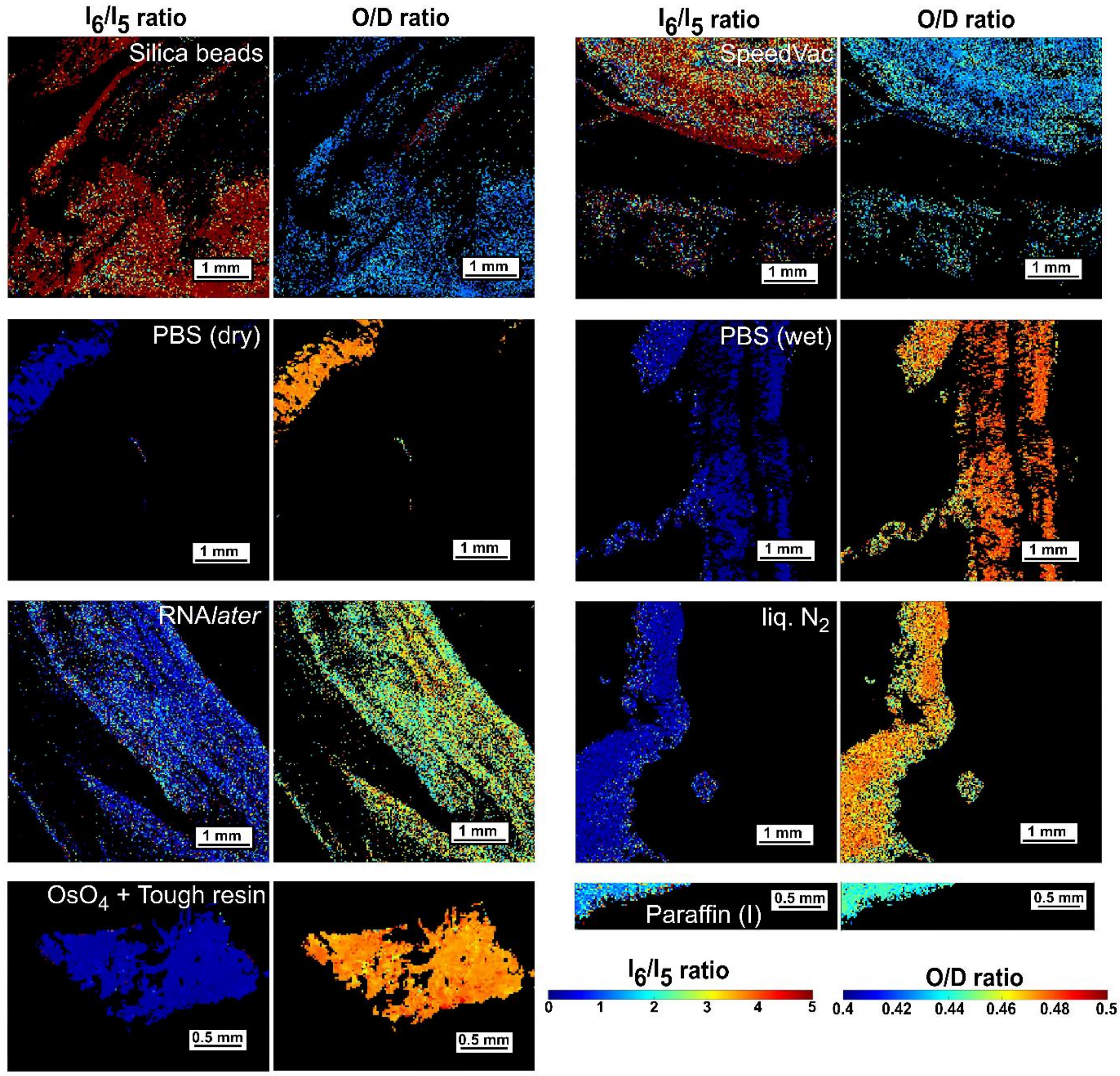

Figure S2 Image plots corresponding to I_6/I_5 ratio (left), linked to dehydration and overlap/*D*-period ratio (right) are illustrated in remaining samples.

S4. Collagen: Orientational analysis

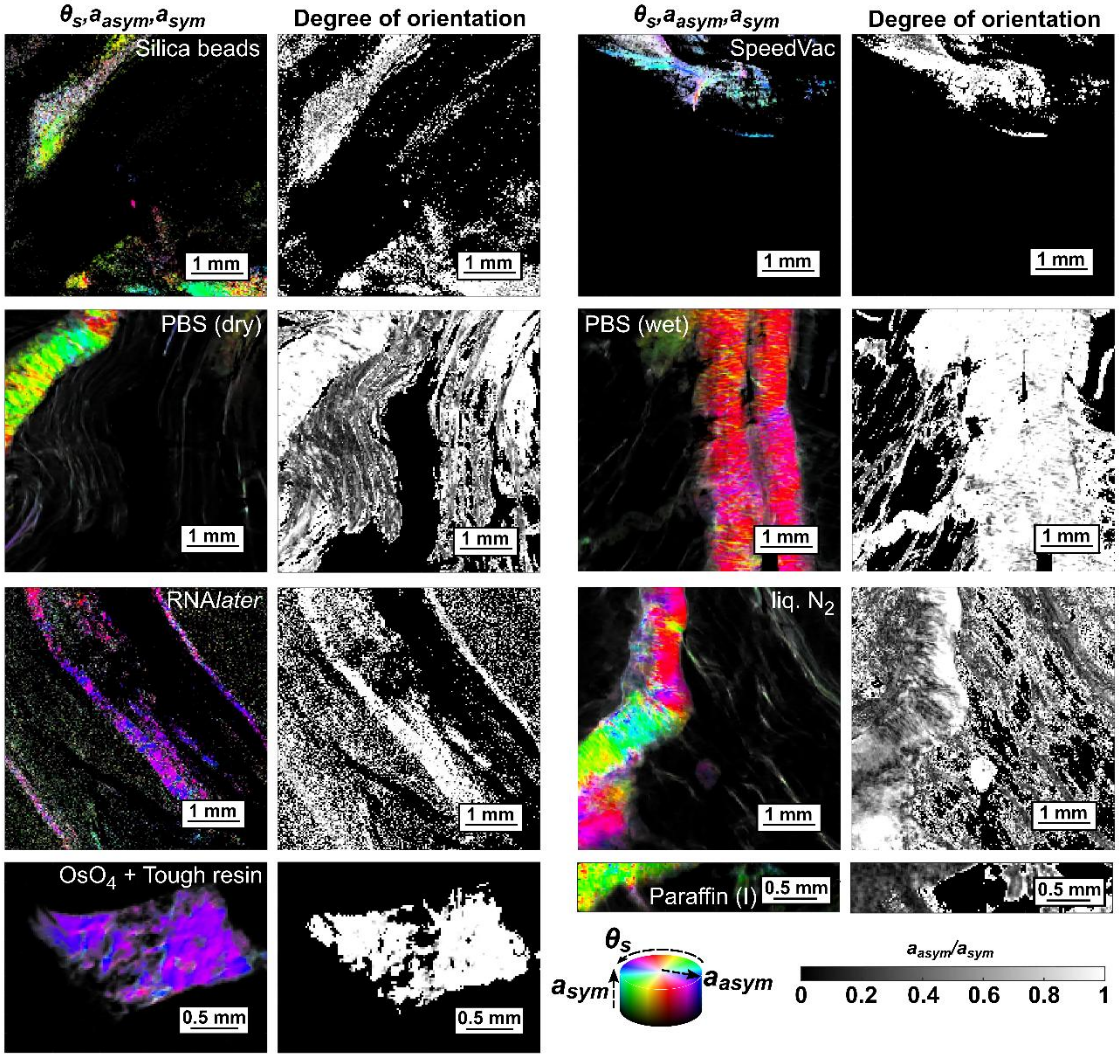

Figure S3 Scanning SAXS images of remaining samples over the q-range = 0.085 nm^{-1}-0.11 nm^{-1}. On the left, images are plotted using an HSV scale where the hue scales with the orientation of scattering (θ_s), according to the colour wheel displayed, the saturation scales with the asymmetric amplitude of scattering (a_{asym}) and the value scales with the symmetric scattering amplitude (a_{sym}) ; on the right, the images illustrate the ratio of the asymmetric to symmetric amplitude of scattering (a_{asym}/a_{sym}), i.e. the degree of orientation, that scales from 0 to 1 for all figures, according to the colorscale in gray.

S5. Myofibril: Ratio of equatorial peak intensities

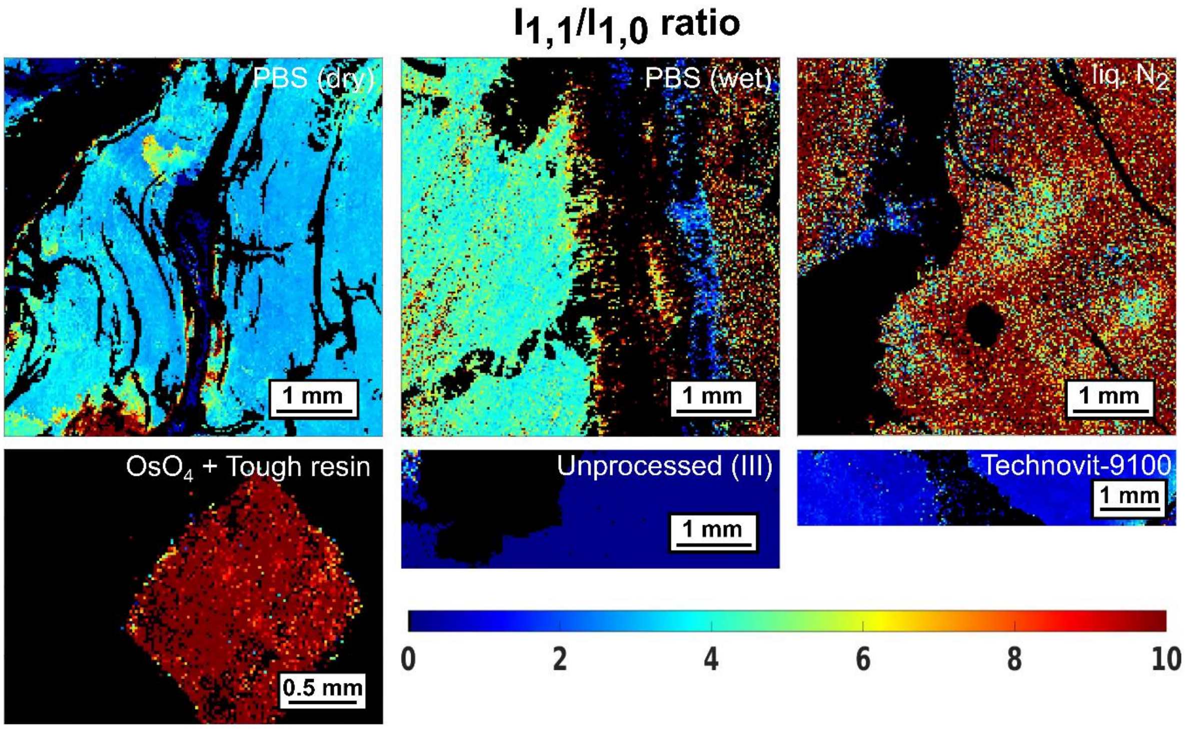

Figure S4 Image plots depicting ratio of equatorial peak intensities in myofibrils: $I_{1,1}/I_{1,0}$ in remaining samples.
